# Ultrasonics and sonochemistry: Editors’ perspective

**DOI:** 10.1016/j.ultsonch.2023.106540

**Published:** 2023-07-31

**Authors:** Sivakumar Manickam, Daria Camilla Boffito, Erico M.M. Flores, Jean-Marc Leveque, Rachel Pflieger, Bruno G. Pollet, Muthupandian Ashokkumar

**Affiliations:** a University of Technology Brunei, Faculty of Engineering, Gadong, Brunei Darussalam; b Montreal Polytechnic, Montréal, Quebec, Canada; c Federal University of Santa Maria, Santa Maria, Brazil; d University Savoie Mont Blanc, Department of Sciences and Mountain Training, Le Bourget du Lac, France; e Université Montpellier, Marcoule Institute in Separation Chemistry (ICSM), Marcoule, France; f Université du Québec à Trois-Rivières, Trois-Rivières, Quebec, Canada; g The University of Melbourne, Melbourne, Australia

**Keywords:** Ultrasonics, Sonochemistry, Nanomaterials, Sonoprocessing, Environmental remediation

## Abstract

Ultrasonic waves can induce physical and chemical changes in liquid media via acoustic cavitation. Various applications have benefitted from utilizing these effects, including but not limited to the synthesis of functional materials, emulsification, cleaning, and processing. Several books and review articles in the public domain cover both fundamental and applied aspects of ultrasonics and sonochemistry. The Editors of the Ultrasonics Sonochemistry journal possess diverse expertise in this field, from theoretical and experimental aspects of acoustic cavitation to materials synthesis, environmental remediation, and sonoprocessing. This article provides Editors’ perspectives on various aspects of ultrasonics and sonochemistry that may benefit students and early career researchers.

## Highlights

• A broad overview of different aspects of ultrasonics and sonochemistry is presented in this article.
• Among the applications covered are synthesis, emulsification, cleaning, and processing.
• Food-related scale-up challenges are discussed.

## 1 Introduction

Hearing the SOUND coming from an alarm clock early in the morning is not something we like. However, all living organisms use sound waves for various purposes. Depending on the frequency, sound waves can affect humans and animals differently. Humans all listen to and enjoy music if the ‘volume of sound’ is appropriate. Hearing loud music from a neighbour’s party in the middle of the night may not be an enjoyable experience. Sound, at a certain frequency range and beyond a certain intensity level, may cause harmful effects on human hearing and health in general. Such issues are of great concern when the frequency of sound waves is within the human hearing range, viz., 20 Hz to 20 kHz. Human ear cannot recognise ‘infrasound’ below 20 Hz or ‘ultrasound’ above 20 kHz. While we may not hear infra- and ultra- sound waves, we are good at using them for selected applications.

**Fig. 1:**
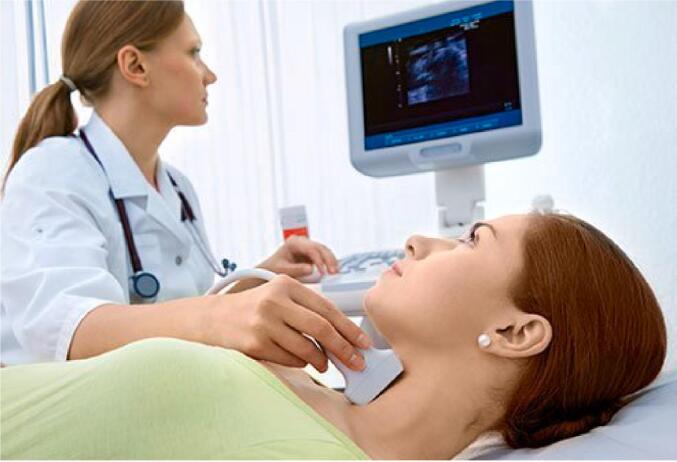
**An ultrasonic scanning equipment for diagnosing thyroid cancer. Image courtesy of WebMed. ©2021, WebMD, LLC. All rights reserved**[11]**.**

In particular, ultrasound (US) has been used in various applications [1], [2], [3], [4], [5], [6], [7], [8], [9], [10]. US is known to the community as a tool for diagnostic medical applications. In Fig. 1, ultrasonic scanning equipment is used to diagnose thyroid cancer [11].

Other applications of US include underwater communication [12], detection of cracks and faults in concrete or steel structures [13], monitoring of food quality [14], etc. Most of these applications are based on the mechanical properties of US. For example, in ultrasonic imaging, high-frequency ultrasound greater than 1 MHz pulses are passed through the human body from the scanning device. These waves are scattered/reflected by the tissue. The intensity of scattered sound waves may vary depending on the nature (normal vs cancerous), shape and distance of the tissue/object. The device then constructs an image by collecting the scattered/reflected soundwaves. This section of the article focuses on using US in chemical and processing applications initiated by a unique phenomenon called acoustic cavitation [15]. It provides the readers with an overview of the fundamental aspects of acoustic cavitation and the current status of ultrasonic applications in key areas such as nanomaterials, biomaterials, industrial processing, etc.

**Fig. 2:**
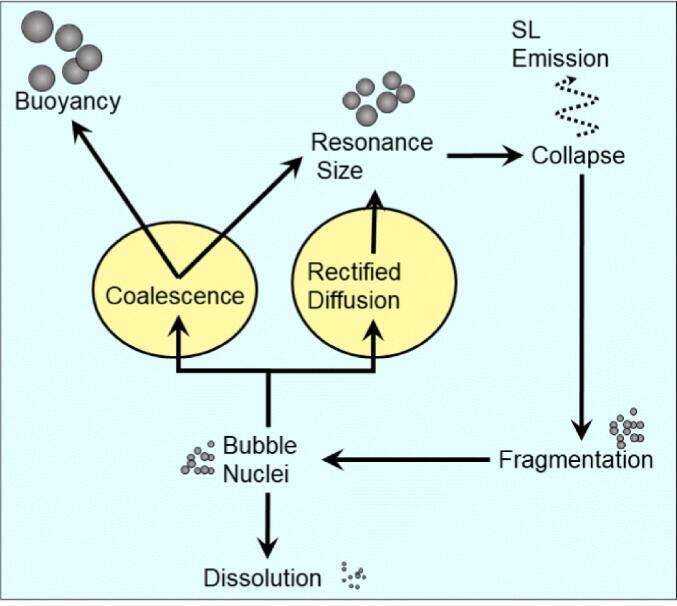
**Schematic representation of acoustic cavitation**[15]**.**

When a liquid is subjected to ultrasonic irradiation, the soundwaves interact with bubble nuclei that are inherently present, ultimately initiating the acoustic cavitation process [15]. Many textbooks and research articles define acoustic cavitation as the US-induced formation, growth and collapse of bubbles in a liquid. However, forming a bubble in a degassed liquid requires enormous energy. For example, breaking intermolecular forces between water molecules to generate a bubble/cavity with a diameter of less than a nanometer requires negative pressure of ∼ 1500 atm [16]. If this is true, no practical applications of US can be developed due to the high energy requirement. There is no need to create a bubble/cavity since most liquids contain dissolved gases (air, for example) that normally exist as bubble nuclei. These bubbles can be grown by ‘rectified diffusion’ [17] or coalescence [18] processes that require much lower energy. Hence, the acoustic cavitation process is the US-driven growth of preexisting bubble nuclei in liquids by rectified diffusion and coalescence processes towards a resonance size range followed by instant growth reaching a maximum size (frequency dependent) and violent collapse. The overall process is schematically represented in Fig. 2.

**Fig. 3:**
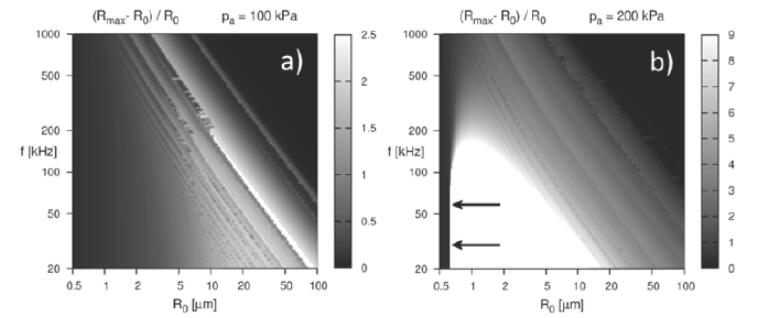
**Evolution of (R_max_-R_0_)/R_0_ (with R_max_ the maximum bubble size and R_0_ the rest radius) with frequency and R_0_ for a driving acoustic pressure of 100 kPa (a) and 200 kPa (b). Resonance sizes are in the white regions; arrows indicate the Blake threshold**[22]**.**

Several review articles describe the rectified growth of bubbles in detail [19], [20]. In short, bubbles in a liquid respond to the pressure fluctuations caused by sound waves and undergo continuous oscillations. During the rarefaction cycle, bubbles grow in size, creating a low-pressure environment within the bubble. This leads to the diffusion of gas/water molecules into the bubble from the surrounding liquid. During the compression cycle, bubbles shrink, resulting in the diffusion of gas/solvent molecules to the surrounding liquid due to the high-pressure environment within the bubble. Since the surface area of the bubble in its expanded state is larger than that in its compressed state, more molecules diffuse into the bubble resulting in the growth of the bubble. This is called the area effect, and a shell effect also operates in parallel, causing a similar growth effect [17]. The second (and maybe main) mechanism of growth is coalescence [21], when two/many bubbles come into contact and fuse/coalesce to produce a bigger bubble. Some bubbles can reach a size at which their growth is particularly strong and collapse violently, the so-called resonance size. This size range depends on the frequency and the applied acoustic pressure p_a_, as seen in Fig. 3. White regions correspond to a very large size difference between maximum expansion and rest radius (radius of the bubble at zero acoustic pressure). A very different behavior can be observed at low (100 kPa) and high (200 kPa) acoustic pressure. At 100 kPa, an increase in frequency leads to a regular decrease in resonant size. At 200 kPa, the image is different: a large interval of resonant sizes is obtained at frequencies of 20–100 kHz, and very strong expansions are obtained already at small rest radii.

In this range of resonance sizes, bubbles can grow to ∼10 R_0_ and then suddenly collapse to a minimum radius of ∼ R_0_/10, leading to a huge concentration of energy, high temperature and pressure and plasma formation. The latter emits light, so-called sonoluminescence (SL).

A very first approximate value of the maximum temperature can be estimated, assuming that the bubble collapse is fast enough to be considered nearly adiabatic. In this approximation, T_max_ is given by Equation (1) [16]:

(1) $$T_{\max}=T_{0}\left\{\frac{P_{m}\left(\gamma -1\right)}{P_{v}}\right\}$$

where T_max_ is the maximum bubble temperature, T_0_ is the solution temperature, P_m_ is the sum of hydrostatic and acoustic pressures, P_v_ is the pressure inside the bubble for p_a_ = 0 and γ is the heat capacity ratio (C_p_/C_v_) of the atoms/molecules present inside the bubble. For example, using T_0_ = 298 K, P_m_ = 2 atm, P_v_ = 0.031 atm and γ = 1.66 (assuming that argon is the only gas present inside the bubble), the T_max_ can be estimated to be ∼12,700 K. Assuming γ = 1.32 (water molecules are present inside the bubble), the T_max_ is found to be ∼ 6,150 K. It can be seen from these calculations that γ plays a major role in controlling the bubble temperature. Equation (1) overestimates the maximum bubble temperature since it assumes adiabatic collapse and neglects endothermic chemical reactions that consume part of the heat energy.

More accurate models [23], [24], [25], [26] have been developed that consider water evaporation and condensation, heat losses due to the gas thermal conductivity, effect of the liquid viscosity, gas ionisation and the formation of a plasma, and chemical reactions. Obtained maximum temperatures are of the same order of magnitude. For instance, for an air bubble in water subjected to 20 kHz US at 20 °C, Yasui et al. [27] calculated a T_max_ of 6300 K for p_a_ = 5 bar and 7300 K for p_a_ = 1.75 bar.

Recent works further improved the model by focussing on non-spherical bubbles, either in a few bubble systems or near a wall, and by combining modelling with observations by a high-speed camera [28]. Several experimental methods are available for the determination of cavitation bubble temperatures. Using comparative rate thermometry in alkane solutions of metal carbonyls sonicated at 20 kHz [29], Suslick and coworkers estimated the gas temperature to be ∼ 5,200 K. The temperature of the thin liquid shell around the bubble is around 1,900 K. Another chemical method was developed by Hart et al. [30], known as the methyl radical recombination (MRR) method. It uses the temperature dependence of rate constants of two competing reactions, namely, the formation of ethane and ethylene from methyl radicals. Using dissolved *tert*-butanol or aliphatic alcohols in water as a source of methyl radicals, measurements were performed in a range of concentrations and derived temperatures extrapolated to zero alcohol concentration, leading to values of 3,400 K at 20 kHz, 4,300–4,600 K at 355 kHz and 3,700 K at 1056 kHz [31].

The estimated temperatures reported in the above studies are time- and volume-averaged temperatures [32]. Hence, the peak temperature reached at the end of bubble collapse, responsible for sonoluminescence (SL), should be much higher than the values reported in these studies. The presence of molecular emissions in SL spectra allows us to derive temperatures that reflect the excitation state of the emitting species. Simulating C_2_ Swan bands emission in the SL spectra of silicon oil [33] and benzene aqueous solutions [34] sonicated at 20 kHz under Ar, Suslick’s group first reported temperatures of 5,075 K and 4,300 K, respectively. These early works assumed the unicity of the temperature, which was later shown not to be a valid hypothesis. Indeed, the plasma formed at bubble collapse is not at equilibrium, and each excited species is characterised by its electronic, vibrational, rotational and translational temperatures. The vibrational (T_v_) and rotational (T_r_) ones can be obtained from emissions simulations. In aqueous ammonia solutions under Ar, for instance, OH rovibronic temperatures were reported around T_v_=9,000 K, T_r_=5,000 K at 20 kHz, those of NH of T_v_=7,000 K, T_r_=4,000 K and at 360 kHz T_v_=13,000 K, T_r_=6,000 K for OH and T_v_=10,000 K, T_r_=2,200 K for NH [35]. It is noticeable that vibrational temperatures are higher at high frequencies, reflecting a higher degree of plasma ionisation. Sharipov et al. [36] measured single-bubble and multibubble sonoluminescence (SBSL and MBSL) spectra of nanodispersed Cr(CO)_6_ suspensions in water and concentrated acids under Ar and derived electronic temperatures from Cr* emission: 8,000 K (SBSL, 26–28 kHz) and 6,500 K (MBSL, 20 kHz) in water, 13,800 K (SBSL) and 11,000 K (MBSL) for H_2_SO_4_, 83% and 16,000 K (SBSL) and 13,500 K (MBSL) for H_3_PO_4_, 74%.

The collapse of cavitation bubbles in a liquid generates several physical forces such as turbulence, microstreaming [37], microjets [38], shockwaves [39], etc. Strong liquid microstreaming around the bubble can be generated during bubble oscillations, as shown in Fig. 3.

**Fig. 4:**
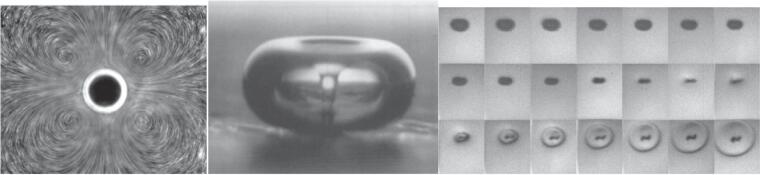
**Microstreaming**[37]**, microjet**[38]**and shockwaves**[39]**generated during acoustic cavitation.**

When a bubble collapses, high-intensity shockwaves are generated that can enhance mass transfer effects leading to an enhanced rate of chemical reactions and particle–particle collisions [40]. Theoretically, the pressure inside spherically collapsing bubbles can reach a few hundred atmospheres [15], [41]. Shockwave generation from a single bubble collapse is shown in Fig. 4, captured using a highspeed camera. When a cavitation bubble collapses asymmetrically, a highspeed liquid jet, travelling at 200 m/s, can be generated. Such liquid jets can increase the porosity of catalytic particles on the surface, leading to enhanced catalytic activity [42].

The strong physical effects generated during acoustic cavitation have been used in several applications, such as emulsification [43], extraction [44], protein denaturation [45], food processing [46] and therapeutic medicine [47]. The mass transfer effects caused by the physical effects of acoustic cavitation have also been used to catalyse chemical reactions and materials synthesis. The plasma generated within cavitation bubbles initiates several (sono)chemical reactions. Sonochemistry is a branch of plasma chemistry similar to discharges in liquids [48]. Chemical reactions can be initiated in both organic and aqueous media. For example, metal nanoparticles could be generated within the cavitation bubbles in organic solvents, such as octanol [49]. When octanol-containing neutral metal complexes such as Fe(CO)_5_ were sonicated, amorphous Fe nanoparticles were generated within the hot zone of the cavitation bubbles. Luche and coworkers have reported several organic sonochemical reactions [50]. When water is used as a solvent (aqueous sonochemistry), H and OH radicals (R1) are generated within cavitation bubbles. It is generally believed that they arise from thermally triggered homolytic dissociation of water molecules. However, recent works pointed out that the mechanism must be more complex and involve electrons from the sonochemical plasma [51].

Molecular products such as H_2_O_2_ and H_2_ are also generated by recombinations (R2 and R3). Several redox reactions could be achieved using these ‘primary’ radicals and these molecules. H atom and H_2_ molecule are reducing agents that can be used for reducing metal ions to generate metal nanoparticles. OH radicals and H_2_O_2_ molecules are oxidising agents and can be used for oxidising various organic compounds. Secondary radicals and other reactive agents could be generated by reacting primary radicals with solutes, for example, with organic molecules (R4), where R stands for an alkyl group or H atom-generated secondary reducing radicals have been used in the production of metal nanoparticles [52]).

H_2_O →H^.^ + OH^.^ (R1)

H^.^ + H^.^ →H_2_ (R2)

OH^.^ + OH^.^ →H_2_O_2_ (R3)

H^.^/ OH^.^ + RCH_2_OH →RCHOH^.^ + H_2_/H_2_O (R4)

In dissolved air, sonochemistry becomes more complex, even in pure water. Both O_2_ and N_2_ react with the formed primary radicals, and a series of reactions occur [53], [22]. For instance, H atoms can be scavenged by O_2_ to form^.^HO_2_ radicals (R5), promoting oxidation reactions. As for N_2_, it can react with O atoms to form NO, leading to HNO_2_ after reacting with OH radical (R6-R9). Further, NO oxidation by O_2_ leads to NO_2_ and the reaction of NO_2_ with OH leads to HNO_3_.

H^.^ + O_2_ →^.^HO_2_ (R5)

O_2_ →))) →2O (R6)

N_2_ + O →NO + N (R7)

N + OH →NO + H (R8)

NO + OH →HNO_2_ (R9)

Where “)))” indicates US.

Nitrogen molecules and atoms can react with H and H_2_ [54], [55], [56], and an almost linear decrease in the yields of H_2_O_2_ and H_2_ in water subjected to sonication at 359 kHz was reported as the N_2_ content in Ar gas increased [56].

The qualitative detection and quantitative estimation of primary radicals have been performed using several analytical techniques. For example, Riesz and coworkers used EPR spin trapping to quantify primary radicals [57]. Terephthalic acid reacts with OH radicals, generating fluorescent 2-hydroxyterephthalic acid that can be quantified by fluorescence spectroscopy [58]. Similarly, salicylic acid can trap OH radicals, and reaction products can be monitored by high-performance liquid chromatography [59]. These techniques only measure OH radicals that escape the bubble interior. Other techniques quantify H_2_O_2_ in solution, like Ti(IV) dosimetry, based on the formation of a yellow complex that can be monitored by UV–Vis absorption spectrometry [60] or the sum of OH and H_2_O_2_ species, like the iodometric method [61]. While the former is H_2_O_2_ specific, the latter requires a catalyst to avoid reactions with the various oxidants.

Reactions R6-R9 illustrate the influence of gas nature on sonochemical activity. The impact of the gas extends beyond its chemical reactivity, encompassing various factors such as the scavenging of radicals produced in the bubbles and reactions between gas molecules. The effects of gas properties on cavitation and sonochemistry have long been recognised. Rare gases, for instance, facilitate a higher concentration of energy at collapse due to their higher polytropic index, lower heat conductivity, and absence of energy dissipation through vibrational excitations and undesired reactions. Furthermore, a lower ionisation potential of the gas promotes plasma formation. Within high-pressure plasmas, reactions primarily occur via 3-body processes, where the third body could be a water molecule or a gas atom or molecule. The reaction cross-section and the lifetime of excited species influence the reactivity. Notably, rare gases possess metastable species, which are excited species with relatively long lifetimes that actively participate in chemical reactions. Research estimates suggest that collisions between metastable Ar* and H_2_O molecules contribute to over 60% of H and OH• production in low-electron density plasmas, which include sonochemical plasmas [62]. Gas solubility is an important parameter that impacts sonochemical processes. Higher gas solubility -reduces surface tension and increases bubble nucleation rate but decreases shape stability [63]. However, the relationship between gas solubility and the number of active bubbles is not straightforward, as increased solubility promotes both nucleation and coalescence, leading to active bubble production and removal. In the case of rare gas mixtures sonicated at 200 kHz, Okitsu et al. discovered a direct relationship between H_2_O_2_ yield and gas solubility [64]. This correlation between bubble number and gas solubility was further supported by Gielen et al. [65], who measured the total bubble volume using the capillary method at 248 kHz for gases such as Ar, air, N_2_, and CO_2_. In the literature, rare gases, particularly Ar, create more extreme conditions during sonochemical processes, often called higher collapse temperatures. This is due to their higher solubility, which enhances degradation through direct pyrolyses, such as the degradation of molecules containing halogens [63]. However, because of its high solubility, Ar may be less effective in breaking down polymer molecules. On the other hand, molecules without halogens appear to be better degraded under air, which promotes the formation of oxidants and radical induced decomposition [63]. Consensus exists that the optimum formation rate of H_2_O_2_ in water, and consequently the highest efficiency in oxidative degradation of pollutants, is achieved with 20–40% O_2_ in Ar [60], [66], [67], [68], [69].

The sonochemical activity can be further enhanced by continuously sparging the solution with gas [60], [70], [71]. Interestingly, Choi et al. found that the gas sparging rate has a more significant impact on activity than the type of gas itself, based on their investigation of Rhodamin B degradation [71]. Consequently, sparging the solution with an Ar-20% O_2_ mixture leads to the highest oxidation rates. Continuous gas sparging offers several positive effects [70], [71]. Firstly, it replenishes the solution with the desired gas, counteracting the degassing effect caused by US. Additionally, it prevents the introduction of air and introduces cavitation nuclei, thereby altering the population of active bubbles. Furthermore, continuous gas sparging deforms or eliminates the ultrasonic field's standing-wave structure, which modifies the active zone's shape and volume. The dissolved gas concentration increased with the gas flow rate [60]. A proportionate relationship between H_2_O_2_ yield and dissolved gas concentration when water was sonicated at 362 kHz under Ar-20%O_2_ flow rates of 20–130 mL/min was also reported [60]. However, this proportionality was not observed for Ar alone [70]. This difference can be attributed to the distinct mechanisms of H_2_O_2_ formation for each gas, with the O_2_ molecule providing intermediate species such as O, HO_2_, and OH. To achieve the optimum positive effect of gas sparging, the positioning of the sparging should be carefully considered [69], [70]. Positioning the sparging point away from the reactor borders but close to the transducer is recommended to supply gas to the largest possible portion of the solution.

It is important to note that the impact of gas can interact with the presence of solutes. For example, in NaCl solutions, studies have shown that at 362 kHz, both H_2_O_2_ and H_2_ yields decrease similarly when Ar was used for sparging. However, when He is used for sparging, the decrease in H_2_ yield is less pronounced compared to the decrease in H_2_O_2_ yield. Additionally, a significant decrease in pH (attributed to the formation of H^+^ ions) was observed specifically when He was used for sparging [72]. This difference in behavior can be attributed to the varying bubble contents caused by the significantly higher solubility of Ar than He. The larger reactive cross-section of Ar also leads to different reaction pathways and probabilities compared to He, thus influencing the observed differences in H_2_ and H_2_O_2_ yields and the resulting pH changes.

**Fig. 5:**
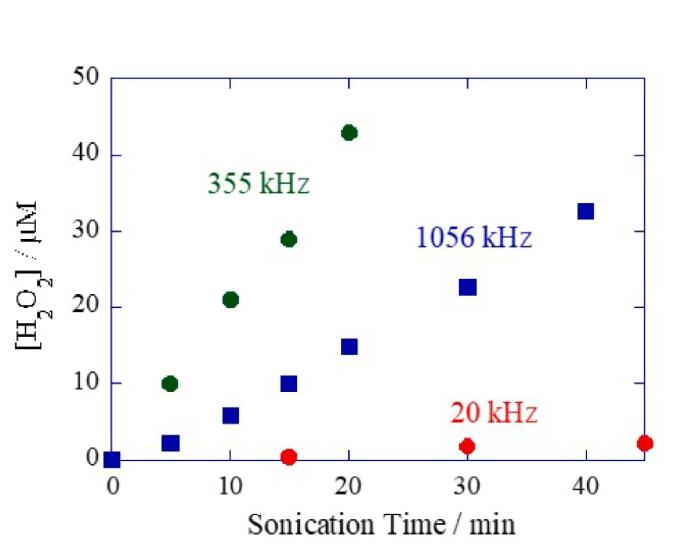
**The amount of H_2_O_2_ (an indirect measure of the amount of OH radicals generated) produced as a function of US frequency**[73]**. H_2_O_2_ yields were measured using the iodide oxidation method, and the acoustic power was kept constant at 0.9 W/cm^2^.**

Finally, the frequency effect is another important experimental factor that needs to be considered in sonochemical reactions. Low-frequency US (primarily 20 kHz) has been known to generate intense cavitation at the tip of the horn. The physical forces of cavitation bubbles are stronger than those of high-frequency US. On the other hand, the amount of radicals generated (responsible for the majority of sonochemical reactions) is the lowest at 20 kHz (Fig. 5) [73]. This makes low-frequency ultrasonic reactors ideal for food processing applications where physical forces are highly useful and radical reactions would be detrimental. Cavalieri et al. [74] have used the emulsifying properties of low-frequency US to synthesise core–shell microspheres for use in biomedical and food applications. High-frequency US generates a relatively higher amount of primary and secondary radicals useful in materials synthesis and environmental remediation. It has been reported that there is an optimal frequency range (200–600 kHz) where the chemical effects of US are found to be maximum. The chemical effects seem to decrease beyond this range: although the plasma characterisation degree was shown to increase with frequency up to at least 3.6 MHz [35], [75], this effect may be counterbalanced by a lower amount of water vapor present inside cavitation bubbles (due to shorter expansion cycle [76]), a decrease in bubble size and possibly to changes in many active bubbles. The frequency effect has been discussed in detail [5].

The chemical and physical effects of acoustic cavitation have been used in various areas and disciplines, from Chemistry to Biomedical and Engineering to Food Processing. This review aims to provide a broad overview of US applications, focusing on the current status and future perspectives. In-depth knowledge on each topic covered could be accessed from references discussed in this review.

## 2 US for environmental remediation

Sonochemical treatments for environmental remediation involve the application of US to treat water, sludge, soil and sediments to remove either organic or inorganic pollutants [77] or separate oily phases [78]. As US generates reactive oxygen species (ROS) that can degrade pollutants, it is classified as an advanced oxidation process (AOP) like ozonation, photocatalysis, oxidation by peroxides, etc. The reactions involving US to generate ROS are the same (R1-R9). If only water is present, ROS are generated by sonolysis (R10 and R11).

H_2_O + ))) → OḢ + OH^−^ (R10)

H_2_O +))) → ½H_2_ + ½H_2_O_2_ (R11)

The main advantage of US for environmental remediation compared to conventional oxidation technologies is the absence of chemicals. They degrade organic pollutants into smaller molecules or, in the best case, into the products of complete mineralization, i.e., H_2_O and CO_2_. Unless the degradation is complete, products might still carry some toxicity. It is, therefore, important to track the environmental toxicity of these components and fate while performing US-assisted laboratory tests, whether in the presence of other AOPs or as a particular degradation method [79].

**Fig. 6:**
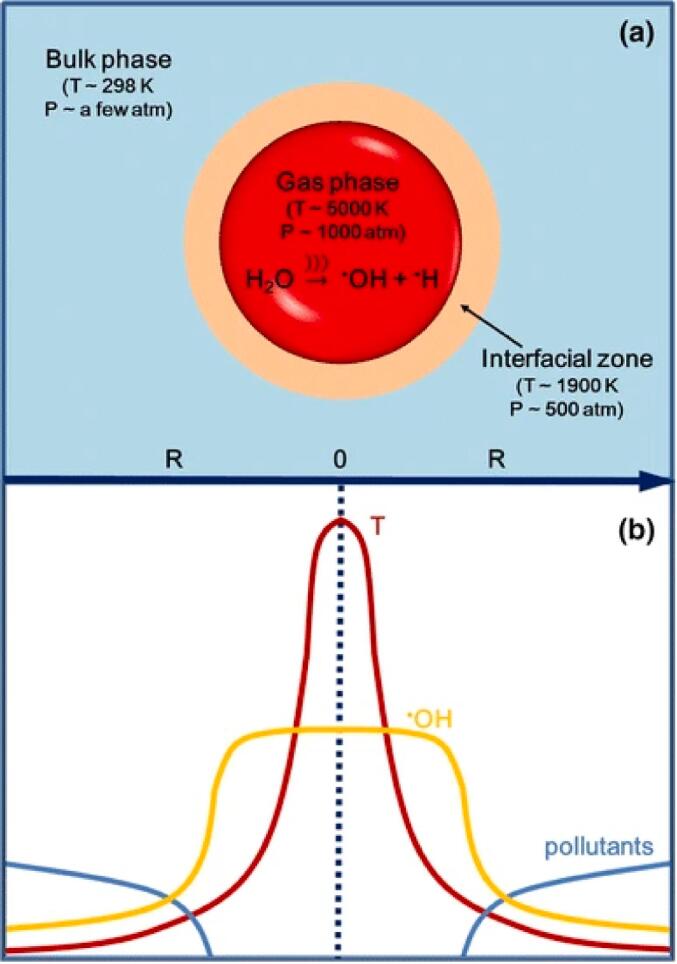
**Schematic of the temperature profile for the US-generated hot spot theory for a cavitation bubble (a) and corresponding profiles of temperature (T), ·OH, and pollutants (b; the vertical axis is arbitrary)**[81]**.**

An organic molecule can degrade in a sonicated system following two pathways. Either heat decomposes the gas and interfacial regions of the cavitation bubbles during the compression phase or at the moment of the collapse, or it is ˙OH radicals or other ROS that degrade it by oxidative cleavage in the gas, interfacial and bulk regions as a few radicals can migrate from the core gas phase to the bulk region [80] (Fig. 6).

The main mechanism of US to degrade pollutants, whether in water, sludge or soils, depends on the formation of ROS, mainly radicals, that form as a consequence of the implosion of the acoustic bubbles. When this latter occurs, hot-spots form with temperatures in the order of several 1000 K and pressures on the order of 100 atm. At these temperatures, the main ROS is ˙OH when the temperature is in the 4000–6500 K range and the O atom when the temperature is above 6500 K [82]. ˙OH may then recombine into H_2_O_2_, which is a ROS as well. However, other species form from the pyrolysis of water vapor when a bubble collapses. When the medium is H_2_O, these also include Ḣ, O_2_˙^-^, HO_2_˙, besides ˙OH, dissolved O_2_, and O atoms [83].

In such a case, the degradation of organic pollutants can proceed through different mechanisms, which can all be referred to as “wet oxidation” (R12-R17) [84]:

RH + OḢ → Ṙ + H_2_O (R12)

RH + O_2_ → Ṙ + HO_2_˙ (R13)

RH + HO_2_˙ → Ṙ + H_2_O_2_ (R14)

RH + OḢ → Ṙ + H_2_O (R15)

R ˙+ O_2_ → ROȮ (R16)

ROȮ + RH → ROOH + R. (R17)

The following sections briefly discuss the main parameters affecting US pollutant degradation in soil, water and sludge. We also provide a perspective on the subject. We cite the comprehensive reviews published, inviting the reader to refer to them for more detailed information about the single methods. Meroni et al. [85] recently reviewed the state of the art of US technologies scale-up to commercial facilities.

### 2.1 Soil remediation

US can be applied to agitate the soil, keep the soil in suspension, and desorb contaminants from sediments [86]. US is usually combined with other treatments in soil environmental remediation as it does not deliver the desired pollutant degradation as a standalone method [87]. These complementary methods may include traditional physicochemical processes, such as soil washing [87], persulfate [88], or more advanced methods, such as advanced oxidation processes (ozone, [86]) and supercritical extraction [89]. Song et al. [87] remediated a model of polluted soil containing phenanthrene concentrations from 0.0125 to 0.05 wt%, using a combination of soil water washing and US at 20 kHz (power unspecified). 69.5% of phenanthrene was removed after 20 min.

**Fig. 7:**
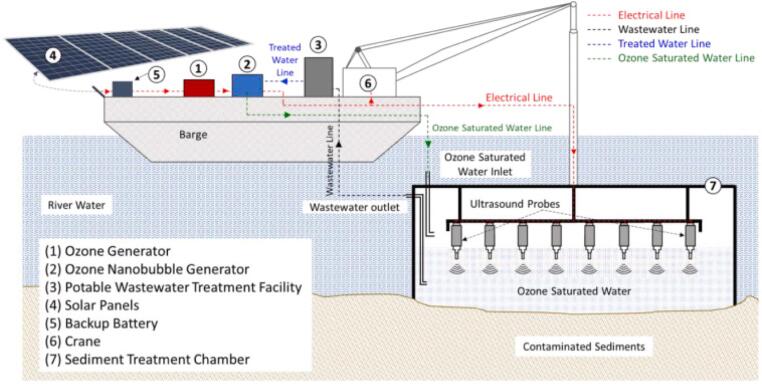
**Field implementation of river sediment combined US and O_3_ treatment**[86]**.**

Aluthgun Hewage et al. [86] coupled US and ozone to remove Cr(III) and P-terphenyl from artificially contaminated river sediments. US was powered as a standalone probe at 20 kHz and 1200 W to treat 80 g of contaminated soil in a 3.5 L chamber with a continuous ozonated water supply. In 240 min of pulsed US (2 min) and 300 L of ozonated water circulation, Cr(III) (4211 mg kg^−1^) and -terphenyl (1875 mg kg^−1^) removal was above 90% when present as single contaminants; however, the removal dropped below 71% when the two contaminants were present together [86]. This system is designed to be scaled up as an in-situ treatment to implement from a barge, whereby the treatment chamber prevents wastewater from contaminating the surroundings. Wastewater is further treated by nanofiltration and subsequent precipitation. The treatment chamber contains a series of US emitters and a generator of O_3_ nanobubbles (Fig. 7).

Castelo-Grande et al. [89] used pre-treated soil samples contaminated with 150–210 ppm of atrazine as a feedstock. 32–35 g of sample were contacted with CO_2_ at 100–245 bar (70 and 80 mL min^−1^) and 30–60 °C (supercritical CO_2_). A 50 W (frequency unspecified) US emitter was glued on the outside wall of the extraction chamber. They reached 91.5 % degradation at 245 bar and 60 °C after 8 h. Cao et al. [90] reviewed the literature on the sonochemical degradation of poly- and perfluoroalkyl substances in soil and examined the effect of power density and US frequency, besides temperature and pressure. They observed that the main positive effect of US in the degradation of pollutants in the soil is due to the increased porosity of the soil and percolation rate enacted by US, which thus promotes mass transfer and accelerates the desorption of contaminants.

Another environmental application of US to solids is the oil recovery from oily solid wastes or sludges [91], [92]. The most important parameters affecting oil separation are US power and hydrophilicity of sludge; a hydrophilic sludge is more prone to let oil separate [93]. However, the variability in the composition and physicochemical properties of oil-contaminated sands, soil and sludges makes it challenging to design a commercial US-assisted process for deoiling. In the available literature, parameters such as wettability between the oil and solid, permeability and porosity are often missing. These parameters are key to investigating the interaction between the solid surface and the oil. These eventually influence the US-assisted deoiling process that likely depends on the characteristics of sand and oil [78].

Mat-Shayuti et al. [92] reviewed the US-assisted processes to clean oil-contaminated sands. They concluded that the mechanisms enacted by US in deoiling sand are still unclear. For instance, it is not yet understood which of the major forces involved (mixing, streaming, macro or micro-shearing, shockwaves or jets generated by acoustic bubbles’ implosion, sand fragmentation, induced temperature increase) drives the oil separation from the sand. They recognize that mathematical modeling, specifically heat and mass transfer modeling, can help scale up oil recovery from contaminated solids with US.

### 2.2 Sludge treatment

Sludge treatment is one application whereby sonoprocessing has been successfully scaled up [85]. US applied to sludge has several purposes, whereby most fall under the category of “sludge disintegration”. US disintegrates sludge particles, creating a more specific surface area for bacteria to access and accelerate fermentation [94]. Further, US ruptures microbial cells, releasing intracellular enzymes [95], thus increasing biomethane yield and reducing waste in general [96]. Processes that are part of sludge disintegration and can be enhanced by US include sludge dewatering, whereby a concentrated, consolidated sludge along with a diluted stream, mostly water, are the products [97], and decontamination from organic pollutants [98].

Concerning US reactor design and operating conditions that seem to work best for sludge disintegration, 20 to 40 kHz, i.e., low range US works best for sludge treatment compared to a higher frequency as bigger acoustic bubbles are closer in size to large soil flocs and interact more [85]. Further, Bandelin et al. [99] compared sonotrodes and flat reactors to treat different types of sludge and quantified the biogas produced after the treatment. They found that energy input being the same, sonotrodes are better for processing viscous waste-activated sludge with high solid content. At the same time, tubular reactors are more suited for less viscous waste-activated sludge. For instance, Oh et al. [98] observed that lighter and more water-soluble polycyclic aromatic hydrocarbons (PAHs) were leached by US preferentially to the supernatant. At the same time, heavier and more hydrophobic PAHs were bound strongly to particles. For a wider overview of sludge sonoprocessing, particularly large-scale applications, the readers could refer to the review of Meroni et al. [85].

### 2.3 Water treatment

In 2012 Eren [100] reviewed the literature on US as a standalone or complementary process to remove dyes from wastewater. The author concludes that all studies are limited to the laboratory due to the scale-up challenges. Indeed, when treating wastewater, the main limitation is the huge power dissipation into the water surroundings. Theerthagiri et al. [101] recently reviewed sonoelectrochemistry for energy and environmental applications. Sonoelectrochemistry couples US and electrochemistry and may provide a synergistic effect to degrade pollutants, possibly leading to higher pollutants degradation than individual methods. These synergistic effects include enhanced electrochemical diffusion, decrease in cell voltage and electrode overpotential, delayed electrode fouling (US “sweeps” electrodes’ surface from contaminants), degassing at the electrode surface as US removes bubbles, and surface activation by metal depassivation by US and thinning of the diffusion layer thickness. Further, the authors conclude that literature data are limited to lab-scale tests. Key parameters to optimise pollutants degradation for water treatment are US frequency and power, irradiation time, US transducer–electrode distance, electrode potential and material, and electrolyte composition [101].

Cao et al. [90] reviewed the literature on the sonochemical degradation of poly- and perfluoroalkyl substances (PFAS) in water. They examined the effect of power density and US frequency, besides temperature and pressure. They observed that while volatile organic pollutants (VOCs) and bicarbonates in groundwater hinder the sonochemical degradation of PFAS, dissolved organic matter (DOM) does not influence it. Further, they conclude that for PFAS, which are highly recalcitrant, combining US with another AOP may work best if the effect is synergistic. They also recognize the challenges in scaling up US technologies, including US emitter erosion, tracking acoustic cavitation in larger reactors, and the presence of several pollutants simultaneously, resulting in competitive degradation [90].

Adding salt ions generally increases the overall degradation rate in the presence of US as it changes the surface tension and ionic strength of the aqueous phase and the concentration of the pollutants at the interfacial region of the cavitation bubbles [90]. These include sulfate [102], persulfate [103], [104], persulfate/chlorite (SO_4_^2-^/ClO_2_^−^) [105], and combined Fe/persulfate systems [106], [107]. In Fe/persulfate system, Fe, besides generating the Fenton system, which acts as an AOP, activates the persulfate ion (SO_4_^2-^) to a sulfate radical (SO_4_^·-^), which oxidizes water contaminants [108]. US can as well promote the reduction of the persulfate ion. Indeed, sulfate radicals (SO_4_^·-^) have a high oxidation potential (2.5–3.1 V vs NHE) and have a longer life span than (ultrasonically generated) ^·^OH radicals [109]. US works in tandem with these oxidants by generating ^·^OH radicals and promoting the formation of sulfate radicals. Adding H_2_O_2_ also results in a synergistic effect with US up to an optimal concentration typical of each system [110]. Beyond this value, ·OH and H_2_O_2_ react to form hydroperoxyl radicals (HO_2_^·^) that are less reactive as oxidizing agents [90].

Anandan et al. [84] recently reviewed US-assisted hybrid water treatment techniques. They conclude that US alone is insufficient to mineralize pollutants quickly. Combining US with other AOPs and/or oxidizing agents (such as H_2_O_2_ or salts mentioned above) is key. However, retrofitting sonolysis to established wastewater treatment plants may bring advantages such as decreasing operating costs and decreasing the usage of other oxidizing chemicals, such as ozone, H_2_O_2_ and Fenton reagents [84]. Wei et al. [81] reviewed the effect of pH on the sonochemical degradation of organic pollutants. For systems where US is present as individual AOPs, pollutants’ degradation generally decreases with increasing pH as ˙OH recombines to H_2_O_2_ at higher pH. Different buffer solutions (e.g., HCO_3_^–^ and CO_3_^2–^) scavenge ˙OH radicals, which also deprotonate at pH greater than 11. For synergistic systems working in tandem with US, the effect of pH on ˙OH radicals is more complex. In general, in the presence of O_3_, the degradation increases by increasing pH; as ^–^OH initiates O_3_ scission, so does the US-UV–H_2_O_2_ system. In the presence of the Fenton reagent, higher pH increases pollutants’ degradation as Fe dissolves at low pH, thus promoting ˙OH in water [81]. However, changing pH continuously to treat water is not feasible for industrial applications.

For water treatment, set-ups at frequencies beyond 100 kHz degrade pollutants more efficiently than the more commercially available 20 kHz equipment [90]. A combination of two frequencies is beneficial for the superimposition principle, according to which sound waves of different frequency interfere constructively and form a standing, greater amplitude wave around the center of the reactor. A wave of greater amplitude leads to faster cavitation bubble growth and more intense cavitational collapse. In a few words, it forms a broader sonochemical reaction field [111], [112]. Increasing temperature also leads to a higher degradation of water pollutants; however, this does not apply to large basins. It might not be practical for closed reservoirs unless heat is produced through renewable electricity.

Sonoprocessing has been successfully applied at the laboratory scale to degrade various organic or inorganic pollutants from water to sand in matrices. While sludge disintegration is one of the successful commercial applications of US at the industrial scale, the huge power dissipation of US in water limits its scale-up for water treatment. Further, when degrading real matrices whereby several pollutants are present, there is competition for oxidation by reactive oxygen species (ROS). At the same time, most examples of complete mineralization are limited at the laboratory scale and to a few pollutants, which is very far from reality. In addition, at the laboratory scale, high-power US is usually delivered in small volumes (high power density), which becomes uneconomical if translated to bigger volumes of commercial applications. Coupling US with other advanced oxidation processes (AOP) has a synergistic effect (beyond additive). Similarly, tweaking the pH accelerates the sonolytic degradation rate depending on the nature of the pollutant. In any case, this requires adding chemicals to the water, which must be separated or neutralized. In general, dual-frequency US equipment (one frequency less than 100 kHz and another greater than 100 kHz) delivers the best degradation rate for various pollutants in water. In contrast, in most cases, lower frequency works best in the case of sludge. The degradation rate increases by increasing US power density until an optimal value whereby the acoustic bubble becomes not transient.

In general, research efforts should aim:

(i) To design reactors with uniform US field distribution. In this case, computational fluid dynamic (CFD) modeling may help.
(ii) To design continuous processes vs batch.
(iii) To find emitter materials and reactor geometries to minimize erosion and corrosion.
(iv) To perform techno-economic calculations to translate the energy consumption of the laboratory tests to large scale.
(v) To track the toxicity and environmental fate of the degradation intermediates, as they could be more detrimental than the parent compound.

## 3 Sonoprocessing

**Fig. 8:**
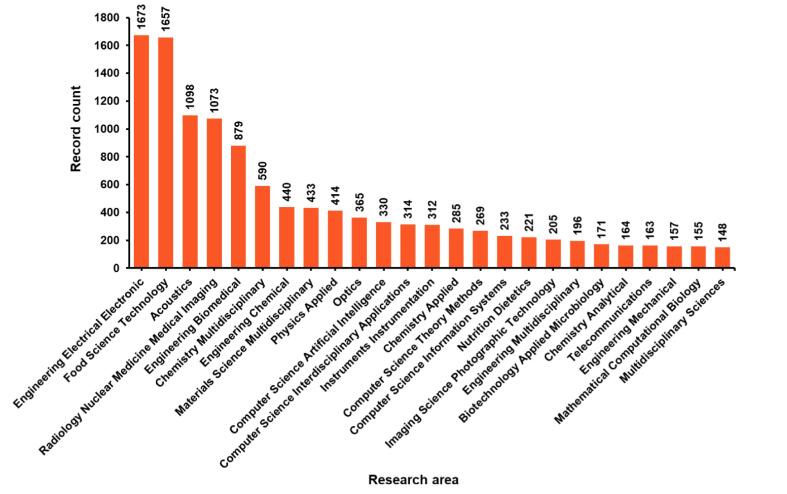
Papers published from 2011 to May 2023. Searched keywords were “ultrasound” and “processing” or “sonoprocessing”. Data extracted from Web of Science, Clarivate Analytics.

Sonoprocessing is a multidisciplinary subject that has been broadly discussed, with over ten thousand studies with the combined topics “ultrasound” and “processing” being featured in Web of Science™ under several research areas over the last twelve years (Fig. 8). Of these studies, the most significant contributions have been in chemistry, food science, technology, engineering, and acoustics.

Although applications on the industrial scale are still scarce, this emerging technology has been growing steadily in process intensification [113]. Processes such as emulsification and separation of emulsions [114], [115], [116], advanced oxidation processes [117], extraction of bioactive compounds and production of biofuels from bioresources [118] have greatly benefitted from ultrasonic effects. Especially in food processing, US has been applied to several procedures, such as the inactivation of microorganisms, separation and emulsification, drying, freezing and thawing, and alteration of physicochemical properties [119], [120], [121], [122], [123], [124]. This growth in applications of US can be related to some advantages of this technology in comparison to conventional processes, such as higher reaction rates, better yields and selectivity, the possibility of using only water as a solvent, milder experimental conditions (enabling extraction/preservation of heat-sensitive compounds), and environmental friendliness [125], [126]. This section will briefly discuss the processes of US-assisted extraction (UAE), emulsification and de-emulsification developed in the last twelve years, focusing on specific applications.

### 3.1 US-assisted extraction (UAE)

Several reviews of interesting industrial compounds have been published in UAE since 2011 to 2021 [118], [127], [128], [129], [130], [131], [132], [133], [134]. Applications include extracting oils, proteins, lipids, dyes, antioxidants, phenolic compounds, anthocyanins, aroma components, and carotenoids [133], [135]. One of the main mechanisms for UAE is the micro-jets generated upon asymmetrical bubble collapse in the vicinity of the matrix [129], [135]. These micro-jets disrupt the cell walls, increasing the mass transfer of solutes into the medium and facilitating solvent penetration into the matrix [135]. Additionally, it was observed that US enhances hydration and swelling on the matrix, further increasing solvent-matrix interactions. This could enable higher yields and faster extractions at milder temperatures and pressure conditions [129].

While US can improve mass transfer and prevent solid clogging in reactors, continuous US presents a challenge due to the associated temperature rise, particularly when utilising high-power inputs ranging from 20 to 100 W [136]. An alternative approach is to employ US waves in a pulsed mode to address this issue. In this configuration, the application of US is alternated with a “silent” period, where no US is applied. This pulsing method offers several advantages, including reduced power consumption compared to continuous US. Furthermore, the authors of the study indicated that while the influence of duty ratio (the ratio of active to silent periods) on process efficiency has been extensively studied, other pulse parameters such as pulse period and load power distribution over a period have received limited attention in research [136].

A popular topic in UAE studies is enhancing antioxidant activity in extracts [134]. UAE of polyphenols from orange peels waste after microwave treatment resulted in a 30% increase in total phenolic compounds compared to conventional extraction, using a 25 kHz bath-type reactor operating at an acoustic intensity of 0.956 W/cm^2^ for 30 min at 59.83 °C [137]. In a study by Rosello-Soto *et al*. [134], different studies on UAE of antioxidants (polyphenols, carotenoids and chlorophylls) from plants, using ethanol and water as a solvent, were compared, and increases in antioxidant capacities of the extracts ranging from 0% and up to 229% were observed. In a different approach, by using sunflower oil as an alternative solvent and a 20 kHz probe type reactor operating at 22.5 W/cm^2^ at 40 °C, it was possible to obtain β-carotene yields from fresh carrots which were comparable to the conventional method in a third of the time (20 min, as opposed to 60 min) [138].

The extraction of pectin, a relevant molecule used in food processing, was also studied under ultrasonic conditions. UAE of pectin from grapefruit was investigated using a 24 kHz probe-type reactor at 200 W for 25 min at 70 °C [139]. Under these conditions, pectin yield was slightly lower (17.92%) than the conventional method (19.16%). However, this result was three times faster at a lower temperature. Additionally, the authors obtained even higher yields when combining UAE with microwave-assisted extraction (31.88%). Another study investigated the differences between pectin extracted from grapefruit peel in UAE and the conventional method [140]. In this study, a 20 kHz probe-type reactor was used for UAE and sonication was applied at 0.41 W/mL of acoustic density for 28 min at 67 °C, in pulsed mode (2 s on: 2 s off). Overall, pectin obtained by UAE showed a lower degree of methoxylation, but higher acetylation and branch chains content, lower molecular weight, viscosity, elasticity and crystallinity, a smaller molecular weight distribution, and higher thermal stability, antioxidant activity and lipase inhibitory capacity [140].

Microalgae have been considered a potential energy source in searching for new biofuels, as they can be mass-produced without competing with human food production and without potable water [141]. In this way, different extraction methods of oil and lipids from this biomass have been developed using US. A probe system operating at 20 kHz and 1000 W for 30 min, containing 5% of dry microalgae, enabled satisfactory oil recoveries compared to the conventional extraction method [141]. A different study developed a cup-horn-like system to extract oil from microalgae [142]. While the conventional method required using a mixture of methanol and CHCl_3_ optimised, UAE conditions enabled comparable yields in shorter reaction times using lower amounts of solvent without the need for CHCl_3_. This was achieved using a 19.5 kHz probe with a 21.5 kHz booster with a working power of 100 W at 50–60 °C [142]. Other compounds in microalgae, such as C-phycocyanin, a photosynthetic pigment, have an important role in industrial applications. In this way, a system was developed to improve cell disruption for C-phycocyanin extraction from dry *Spirulina platensis* [143]. In this system, a 20 kHz probe-type reactor working on pulsed mode (5 s on; 5 s off) at 30% amplitude was applied for 10 min prior to liquid biphasic flotation, resulting in a 95.10% yield [143].

Recently, the synergistic effect of US and supercritical fluid extraction on caffeine recovery from green coffee beans was investigated to improve the process's selectivity and mass transfer [144]. For this procedure, the supercritical CO_2_ was passed through a 1 L extraction chamber containing 185 g of coffee beans, in which a 40 kHz ultrasonic probe working at 90% W was immersed. The probe was operated in pulsed mode (5 min on; 2 min off). After 1 h of treatment, caffeine extraction was twice the amount obtained by the conventional method and extract purity was 10% higher, with higher yields obtained in longer treatments [144].

As seen from the selected applications, using US technology for extraction processes can be an interesting alternative to conventional methods, which usually require high temperatures, long treatment times and potentially toxic solvents. As applications in several areas have been developed, US as a conventional extraction could be implemented. However, there is still much to develop in scaling up to maintain extraction efficiency without compromising other aspects of UAE. In UAE processes, the evaluation of parameters is often limited, with a primary focus on US time and power. Only a few studies have explored other parameters, such as US frequency or the US system used (baths, probes, cup horns, etc.). This indicates a significant opportunity for further research and a deeper evaluation of the effects of US to enhance various extraction processes.

### 3.2 US-assisted emulsification/de-emulsification

The use of US for emulsification and de-emulsification processes has also been extensively studied over the past decade. US-assisted emulsification processes have been especially useful for food and pharmaceutical applications, while de-emulsification processes were mostly used in petrochemical and food processing applications [145], [116]. It is important to note that operational conditions and matrix composition are critical for these processes. For instance, while low frequencies and high intensity are optimum for emulsifying foods and pharmaceuticals, these conditions are also ideal for dehydrating crude oil [100], [115]. In this sub-section, the particularities of US-assisted emulsification/de-emulsification processes are discussed, and some examples have been presented.

#### 3.2.1 Emulsification processes

The ease of operation and cleaning of reactors, allied to the cost- and energy-efficiency of US, have made it a very attractive option for emulsification processes [114]. Although large-scale operations may still be challenging for some applications, this technology enables the production of highly stable emulsions with smaller droplet sizes and narrower droplet size distribution [114], [145]. Additionally, US has the potential to enhance emulsifying properties of surfactants and reduce the number of emulsifiers necessary for the process [114]. These advantages are of special interest to food and pharmaceutical industries due to the possibility of increasing the bioavailability of poorly soluble bioactive compounds, such as essential oils [114].

The physical effects observed at higher intensities and lower frequencies (e.g., 20 or 24 kHz) are responsible for US emulsification processes [114]. High shear forces and micro-jets at the liquid interface are responsible for causing the eruption of droplets of the dispersed phase into the dispersion medium [114], [145], [146], [147]. With continued exposure to US, the intense shock waves and turbulence generated by cavitation further disrupt the droplets in the dispersion medium. This process leads to stable nanoemulsions with droplets within the size range of 20 to 200 nm [114], [146]. This is possible by carefully optimising the equipment parameters. While lower frequencies are ideal for emulsification, a higher power may not necessarily benefit the process since excessive cavitation near the tip of probe-type reactors can cushion the energy transfer to the bulk solution. Additionally, the temperature should be carefully controlled, as its increase can diminish the effect of bubble collapse by reducing the cavitation threshold and potentially harm thermally labile compounds [114].

**Fig. 9:**
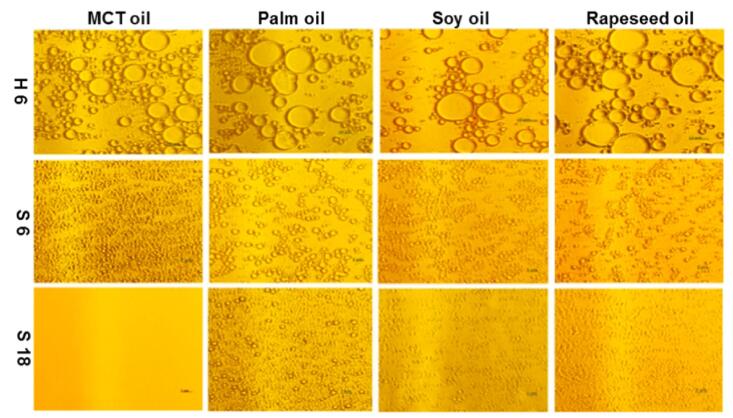
**Images of the obtained O/W emulsions of different oils: medium chain triglycerides (MCT) oil, palm oil, soy oil and rapeseed oil. Images were acquired by optical microscopy. H 6: high shear homogenisation (conventional method) for 6 min, S 6: 6 min of sonication, S 18: 18 min of sonication**[148]**.**

The application of US in pulsed mode has also been investigated. This mode of operation could be an interesting approach to save energy and avoid excessive heating of the medium. As an example, the effects of US on the emulsification of different oils in water using soy protein isolate as an emulsifier were investigated [148]. A probe-type reactor operating at 20 kHz and 40% amplitude in pulsed mode (2 s on: 2 s off) was used, and the temperature was controlled using an ice bath. Intensities of 50 to 55 W/cm^2^, acoustic densities of 1080 and 1620 J/mL and 2 to 18 min treatment times were evaluated. The images of the emulsions after different treatments, obtained by optical microscopy, can be seen in Fig. 9. As shown in this figure, the longer treatment (18 min) for all emulsion types resulted in increased stability and emulsifier absorption. The authors also observed that physicochemical properties varied for different oils. Emulsions containing medium-chain triglycerides presented the lowest zeta potential but were also the most stable, with higher concentrations of absorbed protein [148].

In a different study, the authors reported several advantages to the emulsifying properties of pea protein isolate after US treatment using a probe-type reactor operating at intensities of 57 to 60 W/cm^2^ at 50% amplitude and 39 W for 5 min on pulsed mode (5 s on; 5 s off) [149]. After ultrasonic treatment, the isolate presented greatly enhanced solubility (132% higher), hydrophobicity (52% higher) and emulsifying activity (18 to 27% higher) while also leading to faster absorption when compared to the earlier method [149].

Concerning pharmaceutical applications, the efficiency of sonoprocessing and microfluidization processes was compared to produce aspirin O/W nanoemulsions [150]. This study used a 20 kHz probe-type reactor at 50, 60, and 70% amplitudes at intensities of 55, 66 and 77 kW/cm^2^, respectively, for 100 s. Temperature control was performed using a Rosett cooling cell as the reaction flask submerged in an ice bath. The authors observed that, although samples had to be pre-homogenised for US treatment and physicochemical stability was lower, sonication results were comparable to microfluidization for emulsification of aspirin. On the other hand, to achieve droplet sizes comparable to those obtained by sonoprocessing (below 180 nm), the microfluidiser was significantly less energy-efficient (18-fold) [150].

The incorporation of flaxseed oil into homogenised skimmed milk by US-assisted emulsification was also investigated [151]. Using a 20 kHz probe at 176 W for 3 min, obtaining a stable emulsion containing 7% of oil with an average droplet size of 0.64 mm and surface potential of ≅30 mV was possible. The authors also observed that partially denatured whey proteins acted as surfactants, stabilising the emulsion. Additionally, compared to ultraturrax emulsification under similar conditions, sonication enabled the production of stable emulsions at much higher rates (3 min, as opposed to 20 min for ultraturrax) [151].

A recent review focused on various aspects of ultrasonic emulsification, including emulsification mechanisms, devices, and applications [152]. Indeed, the combination of microfluidics and US offers several advantages, particularly in the control of cavitation phenomena and the intensification of emulsification. By adjusting various flow parameters, such as flow velocity, channel diameter, US frequency, and intensity, it becomes possible to manipulate the cavitation process and enhance emulsification efficiency precisely. Studies have been conducted to understand better the mechanisms involved in microfluidic-US systems. However, it is important to consider the narrow size of microchannels, as it can significantly impact the behavior of cavitation. This effect is known as the channel dimension or wall confinement effect, which refers to the influence of the channel dimensions on cavitation behavior within the microfluidic environment.

#### 3.2.2 De-emulsification processes

For de-emulsification, fractionation or dehydration assisted by US, the main effect responsible for the process is the generation of standing waves [115], [153], [154]. This can be achieved when the distance between emitting and reflecting surfaces is a multiple of half a wavelength, which makes the sound wave reflect upon itself, generating pressure nodes and anti-nodes in regions of destructive and constructive interference, respectively [121]. According to the differences in density, acoustic impedance and compressibility of the dispersed phase, the droplets migrate to the pressure nodes or anti-nodes. Their proximity increases the probability of collision and coalescence [119], [115], [153]. Additionally, droplets of different sizes vibrate differently, favouring these processes [115].

In food processing applications, effects such as cavitation, acoustic streaming, and radical formation can be detrimental to the product and impair the process by promoting secondary emulsification (as exemplified in the previous sub-section) [154]. Hence, frequencies above 400 kHz are usually employed for these processes [116]. However, increasing the amplitude will favour droplet movement within the acoustic field, enhancing de-emulsification [116]. Several applications have been developed in this research area for fractionating fat, olive oil, coconut oil and others [116]. In the dairy industry, US-assisted de-emulsification has been successfully applied to separate fat from milk, as observed in a study by Juliano *et al*. [155]. In this study, the authors subjected recombined milk emulsions and raw milk to different ultrasonic bath configurations and frequencies (400 kHz or 1.6 MHz) for 5 min at 35 °C. Reactor configurations were: i) one transducer in the bottom of the bath (400 kHz and 1.6 MHz), with a reflector at the top of the reaction flask, or ii) two transducers at the sides of the reaction flask (400 kHz), also with a reflector at the top of the flask. Effective separation was observed for both coarse emulsion and raw milk systems. The authors concluded that US could enhance milk creaming in standing waves or heterogeneous wave distributions at higher frequencies [155].

**Fig. 10:**
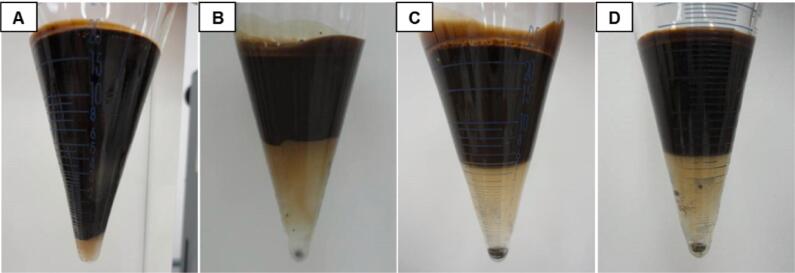
**Pictures of the reaction flasks after US treatment for (A) 1 min at 45 kHz; (B) 15 min at 25 kHz; (C) 15 min at 35 kHz; and (D) 15 min at 45 kHz**[159]**.**

Lower frequencies in the 20 to 120 kHz range are usually preferred for de-emulsification processes in petrochemical applications [116]. Applications such as crude oil and waste oil dehydration have been developed in this area of research [116], [156], [157]. Although cavitation reduces separation efficiency due to secondary emulsification, it has been shown to aid the phase separation process in petrochemical applications [115]. This is because the turbulence caused by cavitation and acoustic streaming could weaken the interfacial film stabilising the W/O emulsion and facilitating contact between dispersed phase droplets [158]. Bath-type reactors operating at different frequencies (25, 35, 45, 130, 582, 862 and 1146 kHz) were investigated for the de-emulsification of heavy crude oil (°API = 19) with different water contents (12, 35 or 50%) [159]. The experiments were performed at 45 °C for 15 min. The highest separation efficiency, approximately 65%, was observed at 45 kHz for the emulsion containing 50% water. After applying different frequencies to the emulsion containing 35% water, the separation can be observed in Fig. 10 [159]. The authors also observed no de-emulsification occurred at frequencies above 45 kHz [159].

The mechanisms for US-assisted emulsification and de-emulsification processes have been widely discussed in the last twelve years. The growing number of studies on US emulsification has been developed mostly in analytical chemistry and food science technology categories. In contrast, studies on de-emulsification were mostly published in multidisciplinary chemistry and food science technology categories. Several applications have been developed, especially for the food, pharmaceutical and petrochemical industries. It is important to note that process parameters are critical in this process and should be carefully optimised, considering the sample matrix and the expected result (emulsification or de-emulsification). On emulsification, an aspect being further discussed is the effect of US on the characteristics of emulsifiers, as it can also influence emulsification efficiency [149]. Furthermore, the challenges in scaling up are still a drawback for US applications either in emulsification or de-emulsification processes at an industrial scale.

An overview of selected applications in UAE and US-assisted emulsification/de-emulsification processes can be observed in Table 1.

**Table 1:** Selected applications in sonoprocessing (US-assisted extraction, emulsification and de-emulsification).

**Aim of the study**	**Type of reactor**	**Experimental conditions**	**Remarks**	**Reference**
***Extraction***				
Extraction of polyphenols from orange peel waste using residual water from essential oil extraction as solvent.	Bath	US frequency: 25 kHz Power: 150 W Intensity: 0.956 W/cm^2^ Time: 30 min Temperature: 59.83 °C	Polyphenol yield was 30% higher using UAE compared to the conventional method.	[137]
Extraction of β-carotene from fresh carrots using sunflower oil as an alternative solvent	Probe	US frequency: 20 kHz Power: 1000 W Intensity: 9.5, 16 or 22.5 W/cm^2^ Time: 20 min Temperature: 40 °C	At 22.5 W/cm^2^, extraction of β-carotene was three times faster than following the conventional method (20 min, as opposed to 60 min).	[138]
Extraction of pectin from grapefruit using acidified water (pH 1.5, achieved by the addition of 0.1 mol/L HCl) as a solvent	Probe	US frequency: 24 kHz Power: 200 W Intensity: n.i. Time: 4 to 30 min Temperature: 50 to 70 °C	Application for 25 min at 70 °C, pectin yield was lower by UAE (17.92%) than by the conventional method (19.16%), but the reaction rate was three times faster and at a lower temperature. A 31.88% yield was possible by combining UAE with microwave-assisted extraction.	[139]
Characterisation of pectin extracted by UAE and conventional method, both using acidified water (pH 1.5, achieved by addition of 0.5 mol/L HCl) as a solvent	Probe	US frequency: 20 kHz Power: 800 W Intensity: n.i. Time: 28 min Temperature: 67 °C	Pectin from UAE had lower methoxylation, apparent viscosity, elasticity, crystallinity, molecular weight and molecular weight distribution, and higher acetylation, branching, thermal stability, antioxidant activity and lipase inhibitory capacity. A probe with a 25 mm diameter and 4.91 cm^2^ surface area was used. The acoustic density of the system was 0.41 W/mL. Sonication was applied in pulsed mode (2 s on; 2 s off)	[140]
Lipid extraction from microalgae using water and butylhydrotoluene as solvents	Probe	US frequency: 20 kHz Power: 1000 W Intensity: n.i. Time: 30 min Temperature: n.i.	Lipid extraction yields by UAE were comparable to those obtained by the conventional extraction method. Maximum oil recovery was approximately 0.21%.	[141]
Oil extraction from microalgae using methanol as a solvent	Cup-horn	US frequency: 19.5 kHz (and 21.5 kHz booster) Power: 100 W Intensity: n.i. Time: 2 to 18 min Temperature: 50–60 °C	UAE reduced the amount of solvent, and comparable yields to the conventional method were obtained in shorter times.	[142]
Extraction of C-phycocyanin from *Spirulina platensis* using phosphate buffer as a solvent	Probe	US frequency: 20 kHz Power: 750 W Intensity: n.i. Time: 10 min Temperature: n.i.	A 95.10% recovery and a purification factor 5.25 were obtained when UAE was used as a pre-treatment for extraction using liquid biphasic flotation. Sonication was applied in pulsed mode (5 s on; 5 s off), and amplitude was set at 30%.	[143]
Caffeine extraction from green coffee beans using UAE and supercritical fluid extraction	Probe	US frequency: 40 kHz Power: 90% W Intensity: n.i. Time: 1 h Temperature: n.i.	Caffeine yield was doubled compared to the conventional method (a total extraction of 63.1% was obtained after 4 h of treatment). A purity of 10% higher was reached in the extract from the proposed method. Sonication was applied in pulsed mode (5 min on; 2 min off).	[144]
***Emulsification***				
Investigation of the influence of different oils (medium-chain triglycerides – MCT, long-chain triglycerides, palm, soybean and rapeseed) in emulsification using soy protein isolate as an emulsifier	Probe	US frequency: 20 kHz Power: n.i. Intensity: 50 to 55 W/cm^2^ Time: 2 to 18 min Temperature: n.i.	Longer sonication (12 and 18 min, acoustic densities of 1080 and 1620 J/mL) led to higher stability and protein absorption for all emulsions. At 18 min, MCT emulsions had lower droplet size (0.05 mm), higher stability and absorbed protein but lower zeta potential. Sonication was applied in pulsed mode (2 s on; 2 s off), and amplitude was 40%. A probe with 0.636 cm of diameter was used.	[148]
Study of the structural, interfacial and emulsifying properties of pea protein isolate treated with US	Probe	US frequency: 20 kHz Power: 39 W Intensity: 57 to 60 W/cm^2^ Time: 1 to 5 min Temperature: n.i.	At 5 min, protein had higher solubility (132%), hydrophobicity (52%), emulsifying activity (18–27%) and capacity (11%), faster absorption, and lower susceptibility to lipid oxidation. A probe with a 12 mm diameter was used (25 mm depth). Sonication was applied in pulsed mode (5 s on; 5 s off), and amplitude was set at 50%.	[149]
Comparison of US and microfluidization parameters on the production of O/W nanoemulsions containing aspirin	Probe	US frequency: 20 kHz Power: 1000 W Intensity: 50, 56 and 77 kW/cm^2^ Time: 10 to 100 s Temperature: 30 °C	US was more energy-efficient than microfluidization, but samples had to be pre-homogenised before treatment. Smaller droplets were obtained at higher amplitudes. Amplitude was evaluated at 50, 60 and 70%.	[150]
Incorporating different amounts of flaxseed oil (7–21%) in pasteurised homogenised skimmed milk.	Probe	US frequency: 20 kHz Power: 176 W Intensity: n.i. Time: 1 to 8 min Temperature: 22.5 °C	At 3 min, emulsions with 7% of the oil were stable (9 days, 4 °C). A probe 12 mm in diameter was used. Partially denatured whey proteins aided in emulsion stabilisation. The droplet size was 0.64 mm, and the surface potential was ≅30 mV. Compared to Ultraturrax, US was faster in producing stable emulsions.	[151]
***De-emulsification***				
Fractionation of fat from fine and coarse recombined milk emulsions and raw milk	Bath	US frequency: 400 kHz or 1.6 MHz Power: 1.6 W (400 kHz) or 0.35 W (1.6 MHz) Intensity: n.i. Time: 5 min Temperature: 35 °C	Creaming was less pronounced for the fine emulsion. Separation was possible in coarse emulsion and raw milk depending on the frequency and reactor configuration (transducer at the bottom or both sides). Acoustic density of the system varied from 10 to 25 J/m^3^.	[155]
Dehydration of heavy crude oil synthetic emulsions containing 12%, 35% or 50% of water	Bath	US frequency: 35 kHz Power: 160 W Intensity: n.i. Time: 15 min Temperature: 45 °C	Temperature necessary for the process was lower than that used in conventional methods. Up to 65% of water was removed for emulsions with 50% of water. The acoustic density of the system was 19.2 W/dm^3^.	[158]
Dehydration of heavy crude oil synthetic emulsions containing 12%, 35% or 50% of water	Bath	US frequency: 25, 35, 45, 130, 582, 862 or 1146 kHz Power: 160 W Intensity: n.i. Time: 15 min Temperature: 45 °C	De-emulsification was efficient for frequencies of up to 45 kHz, with water removal yields of up to 65% for the emulsions containing 50% of water. No de-emulsification was observed above 45 kHz.	[159]
n.i.: not informed US: US				

### 3.3 Trends for sonoprocessing

**Fig. 11:**
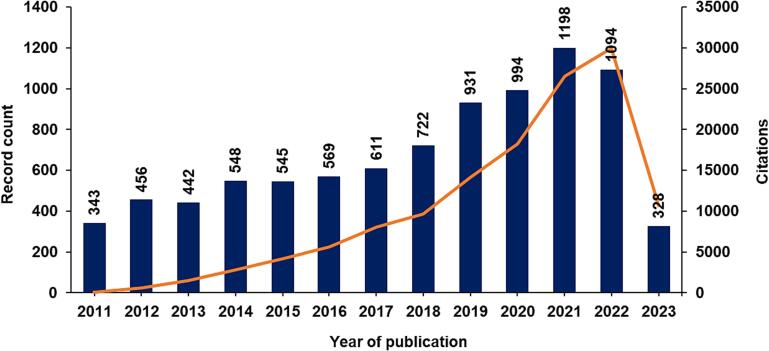
Studies on sonoprocessing published from 2011 to May 2023. Searched keywords were “ultrasound” and “processing” or “sonoprocessing”. Data extracted from Web of Science, Clarivate Analytics.

Sonoprocessing is a fast-growing US research field, as seen in Fig. 11. This is due to the several advantages of this emerging technology in process intensification, exemplified throughout this section. This growth is even more pronounced in the food processing field.

However, as indicated before, a general challenge in sonoprocessing applications is the lack of studies regarding process scale-up. For this challenge to be overcome, further studies must be developed on manufacturing more efficient equipment (such as more robust transducers and systems with uniform energy dissipation) [160].

## 4 Nanomaterials

The non-traditional route of US application as a simple, rapid and easy technique in the generation and modification of conventional bulk or microstructured and nanostructured materials is a widely and intensively investigated area, especially in the last two decades for a range of technological applications such as catalysis, biomedicine, energy storage, hydrogen storage. Top-down and bottom-up are the two common strategies for synthesising bulk and nanomaterials, including various techniques such as ball milling, spray pyrolysis, CVD, PVD, epitaxy, etc. The resultant physical and mechanical effects of cavitation are responsible for the observed outcomes. Optimised ultrasonic conditions are necessary to obtain the desired material with the targeted attributes.

In the case of existing materials obtained by solvent/solution-based techniques such as co-precipitation, solvothermal, hydrothermal, etc., US is employed to assist them. Thus, using US, either the conventional technique could be assisted (intensified), or the toxic precursors and solvents utilised in these techniques could be replaced with simple precursors and greener solvents. US route demonstrates advantages in the reactions involving materials synthesis, such as reducing the reaction time, improving the yield and purity, occurring at ambient/mild conditions, etc. Besides, it has good control in the following: size, homogeneity (monodispersity) (prevents agglomeration), crystallinity, surface area, morphology, stability, etc., leading to interesting physical, chemical, electrical, optical, and photochemical properties of the generated materials. Although synthesis is targeted using US, modification (coating, deposition, immobilisation, decoration, exfoliation) and materials formulation are also exploited.

The ultrasonic parameters controlling the obtained materials depend on frequency, amplitude (intensity), and solvent characteristics such as vapour pressure, surface tension, viscosity, sonication time, precursor concentration, pulse on/off mode, etc. Commonly low frequency (100 kHz) and high-intensity US are employed through bath or probe systems to synthesise materials; however, US systems employing more than 100 kHz have been utilised in a few cases.

Cavitation threshold, a minimum US intensity required to generate transient cavitation, depends on the characteristics of the medium. The short bubble collapse time and very fast kinetics during transient cavitation limit the nuclei from growing, resulting in the smaller size of nanomaterials. Besides, rapid cooling makes crystallisation difficult, leading to amorphous materials. Again, the formed nanoparticles could decrease the cavitation threshold since they act as nucleation centres. Bubbles generated through cavitation act as reactors. Their asymmetric implosion leads to various physical effects of microstreaming; microjets of high speed and shockwaves of high intensity induce effective stirring or mixing or agitation and enhance local heat and mass transfer, which could reduce the particle size, and agglomeration and change the morphology. Nucleation and crystallisation times are accelerated using US. In the case of reactions leading to the synthesis of materials using US, three possible sites have been proposed where the precursors could reach and expose: (1) Interior of the bubble where extreme conditions of temperature and pressure exist once the bubbles collapse (2) Interface between the bubble surface and surrounding bulk liquid where moderate conditions exist (3) Bulk liquid where less extreme or ambient conditions exist. Thus, depending on the volatile nature of the precursors, they can reach any of these regions, i.e., entering the bubble to expose it to the intense conditions of bubble collapse, reaching the interface, or staying in the bulk liquid. Many materials in bulk and nano forms, such as metals, metal oxides (titania, alumina, ZnO), sulphides, selenides, alloys, composites, novel carbon, etc., have been obtained using US. In this review, the focus is only on the very recently published papers.

**Fig. 12:**
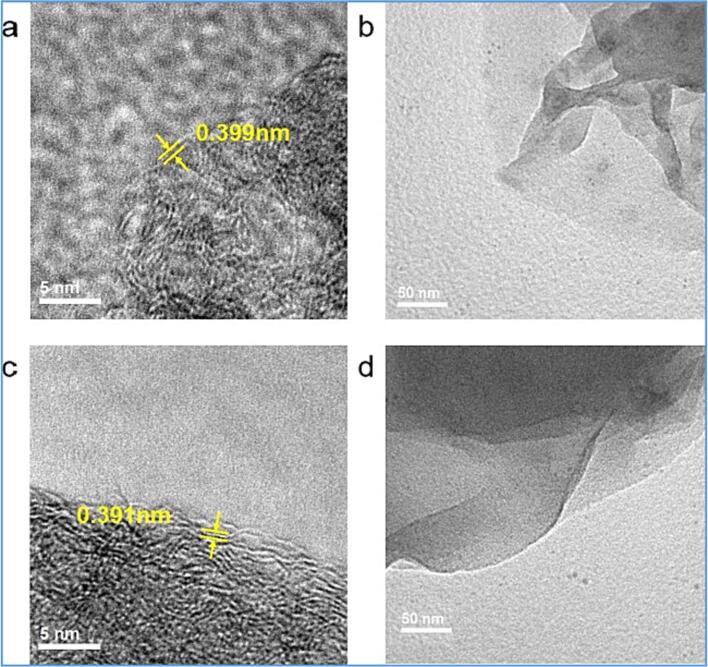
HR-TEM micrographs of GO sheets obtained from sonication in NMP (a and b) and DI water (c and d).

An US-assisted mechano-chemical cracking method has been proposed to produce graphene oxide (GO) [161]. In this study, Miscanthus, an energy crop, was first used as a carbon precursor, pyrolysed at 1200 °C and then subjected to edge-carboxylation via ball-milling in a CO_2_-induced environment. The resultant functionalised biochar was then ultrasonically exfoliated in N-Methyl-2-pyrrolidone (NMP) and DI water to form GOs. It led to good quality and uniform GOs (8–10% monolayer), with up to 96% having three or fewer layers. Small amounts of graphene quantum dots were also observed. The authors proposed that NMP could effectively penetrate and intercalate between pyrolytic biochar layers leading to more complete GO sheets with a lower degree of the defect (lower I_D_/I_G_ ratio). Then, the role of ultrasonication is to effectively promote and propagate the mutual repulsion of functional groups of like-charges, causing a better exfoliation rate than in the absence of sonication. Fig. 12. shows the HR-TEM micrographs of GO sheets obtained from sonication in NMP and DI water.

US-assisted nanocrystalline Ni–W alloy coatings with Ni_17_W_3_ composition were deposited on a copper substrate, and preferential orientation in the plane (2 2 0) was noticed [162]. A decrease in microcracks due to acoustic cavitation-assisted diffusion was observed. Notably, coating produced at 50 °C without thermosonication exhibited the highest polarisation resistance (20.50 kΩ cm^2^). The anti-corrosion property of the generated Ni–W alloy was also examined, and corrosion resistance was not improved by subjecting it to thermosonication at high temperatures.

Electrodeposition is very useful in the fabrication of novel alloys and nanocomposites. Using sonoelectrochemical modes for depositing composite coatings showed high practical potential since US can promote the deagglomeration of second-phase particles in the electrolyte and, consequently, provide fine dispersion of particles in the metal matrix. In liquid media, ultrasonic cavitation decreases the thickness of the diffusion layer and improves mass transport. Cu-Sn-TiO_2_ nanocomposite coatings were electrodeposited under mechanical and ultrasonic agitation (26 kHz and 32 W/dm^3^) [163]. The effect of TiO_2_ nanoparticles and current density on the structural and antibacterial properties was investigated. A comparative study on the effect of mechanical and US agitation on the properties of the obtained nanocomposite coatings was performed. Ultrasonic-assisted electrodeposition significantly improved the surface morphology and distribution of the TiO_2_ particles over the surface. The average roughness of the coatings was 69–73 nm and 45–62 nm for the mechanical and ultrasonic agitation modes, respectively. The nanocomposite Cu-Sn-TiO_2_ coatings formed by ultrasonic-assisted electrodeposition exhibited excellent antimicrobial properties against E. coli bacteria.

Metal-matrix self-lubricating composites (MMSC) containing metal chalcogenides (e.g. MoS_2_, NbSe_2_) as solid lubricants fabricated by powder metallurgy have been widely applied in sliding electrical contact (e.g. brush-slip) for many years. Since ultrasonication proved to be an efficient top-down technique and was explored to obtain NbSe_2_ micro/nanoparticles using single-crystal NbSe_2_ flake as a precursor in ethanol, a mechanical method was employed for comparison [164]. US-assisted exfoliation without aging facilitated the formation of NbSe_2_ micro/nanoplatelets with sizes of 0.1 μm to 25 μm and nano-whiskers with 100 nm diameter and 1∼3 μm in length. Mechanical exfoliation produced NbSe_2_ microplatelets with typical sizes of 1 μm to 30 μm and thicknesses less than 2 μm. The above-produced NbSe_2_ micro/nanoparticles using US without ageing exhibited excellent lubricating properties with low friction coefficient (0.3), mild wear, and longer wear lifetime (120 min) compared to mechanically exfoliated NbSe_2_ microplates (10 min).

Delamination of layered-MOF (MAMS-1) to obtain MOF nanosheet using a more sustainable and efficient deep eutectic solvent (DES) as an alternative in the presence of US than using the conventional organic solvents was reported [165]. Under sonication in the presence of DES as a solvent via poly(vinylpyrrolidone) (PVP) surfactant assistance, the highest exfoliation rate of MAMS-1 was up to 70%, with two host layers achieved.

A new nanohybrid of copper oxide and zinc antimonate was designed using an ultrasonication-assisted homogenous magnetic stirring approach [166]. This combination enhanced the nanostructured electrode's electrical conductivity and charge storage capacity, essential for supercapacitor application. The fabricated nanohybrid electrode material exhibited exceptional electrochemical performance by delivering a maximum specific capacitance of 257.14 F/g at a current density of 12.5 A/g. The nanocomposite also showed high cycling stability of 102.0% even after 2000 cycles at a current density of 10.0 Ag. These exceptional electrochemical characteristics of CuO/ZnSb_2_O_6_ nanocomposites have been proposed due to their dual nanorod morphology, the influence of ultrasonication on non-aggregated nanocomposite formation, the presence of more number of electrochemical active sites, and their synergistic interactions. Notably, ultrasonic waves could disperse the nanorods of CuO and ZSO, thereby promoting Brownian motion.

Using ultrasonication, a 2D-WS_2_ nanosheet with approximately ten layers of thickness of about 7.5 nm and an average lateral size of 100–130 nm was prepared by liquid-phase exfoliation (LPE) [167]. Sonochemical treatment was done using WS_2_ powder and 35 vol% ethanol/water to obtain WS_2_ sheets using continuous ultrasonic bath irradiation (40 kHz, 100 W) for five days. 2D-WS_2_/WO_3_ heterojunction was then built to study its PEC (photoelectrochemical) performance before calcination (WO_3_/WS_2_-90) and after calcination (WO_3_/WS_2_-450). WO_3_ nanoplates for the electrode were synthesised on tungsten foil assisted by US. The obtained electrode showed compact and uniform films easily grown on the entire surface of the W substrate. WO_3_/WS_2_-450 plate-like structure prepared with ultrasonication increased charge separation and reduced recombination of the photogenerated carriers. The fabricated WO_3_/WS_2_ electrodes have been proposed as promising candidates as a photoanode for PEC cells.

The development of antibacterial textiles (antibacterial coatings on textile materials) is one of the new directions. They are beneficial in preventing the possible spread of nosocomial infections (HAI, Healthcare Acquired Infections) in healthcare facilities. For this development, uniform coating, avoiding the accumulation of solid particles, and ensuring the particles' complete attachment on the fibre's surface are important. The velocities of nanoparticles accelerated by the surface of a collapsing cavitation bubble can reach 400 m/s, ensuring tight adherence of the directed particles to the textile surface. Also, it is easy to achieve the required velocities with nanoparticles as they possess a smaller size and less mass than bulk particles. Thus, US can direct and fix nanoparticles on the targeted matrix. Earlier, it has been noted that the separation of nanoparticles occurs in a small amount from the textile during the washing process. However, the coatings obtained through the US route (22.0 ± 0.5 kHz and 1,000 W) were observed to withstand up to 70 wash cycles [168]. This method was developed using a sol–gel method for coating textiles with antibacterial TiO_2_/ZnO nanoparticles using titanyl sulphate and zinc nitrate hexahydrate, suitable for a roll-to-roll application. It has also been tested on a semi-industrial scale. The coated fabrics showed a suppression level of E. coli of more than 99.99% and an antibacterial activity of more than 1.8. Hence, they can be used to prevent the spread of nosocomial and other infections.

2-D graphene has been intensively considered in preparing graphene/polymer composites. In a greener way, without adding any exciter, no solvent, but only with US and stirring, has been proposed to obtain PMMA and Graphene/PMMA composites [161]. Authors have found a threshold (150 W/cm^2^) and optimal ultrasonic intensities (225 W/cm^2^) for initiating ultrasonic polymerisation to obtain PMMA in the supercritical CO_2_ system. Following this, Graphene/PMMA composites were also attempted using US. The uniform distribution of radicals has been noted through ultrasonic excitation, which did not generate unsaturated double-bonded polymers. The electrical conductivity of the ultrasonically obtained composites (graphene content of 1 wt%) increased to 1.13 × 10^-1^ S/cm, better than that obtained through the conventional *in situ* polymerisation method.

In nanomaterials synthesis, ultrasonic microreactors with uniform energy dissipation have emerged as a promising technology. By combining US with precise control of energy distribution, these microreactors can improve synthesis efficiency, reduce reaction times, and improve product quality. They are particularly useful in nanomaterials synthesis, where precise control of particle size, morphology, and composition is crucial. Traditionally, nanomaterials have been synthesized through slow, energy-intensive processes that result in broader particle sizes and limited control of nanoparticle properties. On the other hand, the advantages of using ultrasonic microreactors are as follows. (a) The increased mass and heat transfer: US can facilitate the mixing and mass transfer of reactants in a reaction medium, resulting in a more uniform reactant distribution and more efficient heat transfer, which can significantly speed up the reaction. (b) Reduced particle aggregation and size distribution: Due to the controlled energy dissipation within microreactors, particles do not aggregate and nucleate uniformly, resulting in narrower size distributions and improved homogeneity of final nanomaterials. (c) Scalability and reproducibility: Ultrasonic microreactors can be scaled up and used to synthesize nanomaterials reproducibly, making them suitable for industrial applications. (d) Eco-friendly approach: By maximizing energy efficiency and reducing reaction times, ultrasonic microreactors contribute to a more sustainable synthesis process, thereby reducing the environmental impact of the process.

Using ultrasonic-assisted continuous synthesis, steric capping agents were not required to produce Ag/g-C_3_N_4_ nanocatalysts. In a coiled flow inverter microreactor (CFIR), Ag/g-C_3_N_4_ nanocatalysts were synthesized using the chemical reduction method. In the CFIR, ultrasonic irradiation was used to nucleate and grow nanoparticles. After synthesizing nanocatalysts, their photocatalytic performance was evaluated for water splitting under visible light [169].

In the context of nanomaterials synthesis, especially biomaterials, it is crucial to consider the potential effects of the synthesis process, including US, on the material's integrity and properties. In particular, the mechanical forces induced by US can be detrimental to biomaterials. Therefore, it is crucial to classify different types of materials based on their response to such synthesis conditions. When exposed to even low mechanical or shear stress levels induced by US, highly sensitive materials can be severely damaged or degraded. This category includes biomaterials such as proteins, enzymes, lipids, and delicate organic nanostructures. In high-intensity US, these materials can be denatured, aggregated, or fragmented, losing functionality and biological activity. Consequently, ultrasonic synthesis may not be appropriate for all types of biomaterials. Therefore, alternative synthesis methods or modifications to the ultrasonic process are recommended when dealing with sensitive biomaterials to avoid irreversible damage.

Researchers may adopt the following strategies to address concerns regarding biomaterial sensitivity during ultrasonic synthesis. (a) Lowering ultrasonic intensity: reducing ultrasonic power levels to minimize shear forces without sacrificing sufficient energy to maintain efficient reaction kinetics is the best method for minimizing shear forces. (b) Shorter ultrasonic exposure time: By limiting the duration of ultrasonic exposure, sensitive biomaterials will be less affected by cumulative shear. (c) Temperature control: When ultrasonic reactions occur, cooling systems should be employed to prevent excessive heat from damaging biomaterials. (d) Protective additives: Stabilizing agents or protective additives are added to protect biomaterials from shear-induced damage during their synthesis.

Researchers can achieve a balance between efficient nanomaterial synthesis and preservation of the integrity and functionality of biomaterials by classifying materials based on their sensitivity to US-induced shear and implementing appropriate strategies. With this approach, nanomaterials with tailored properties will be developed for various applications, including biomedical, environmental, and energy-related ones.

US is very powerful and promising as a process intensification technique in the materials area. The cavitation bubbles act as nanometric reactors, and their implosion generates extremely high temperatures and pressures, initiating various physical and chemical changes. Large scale application of US towards materials is not fully realised yet, a key factor to be considered to exploit its fullest potential. In addition to ultrasound cavitation, its counterpart, hydrodynamic cavitation, could play a significant role, especially in a large-scale generation. Although the resultant bubble collapse intensity from hydrodynamic cavitation is low compared to US, developing this technique on a large scale is comparatively easy as it requires a tank, pump, control valve, and pipelines. Besides, its maintenance is easy. Since it is powerful in energy efficiency, it could be explored to achieve the desired physical effects (mixing, dispersion, extraction, cleaning, homogenisation, deposition, coating, etc.) necessary for the materials synthesis and formulation. In the next-generation novel engineered materials, US is expected to play a significant role; overall, US provides endless possibilities in the materials area.

## 5 Sonoelectrochemistry

*Sonoelectrochemistry* is the use of power US in electrochemistry, offering many advantages, including [170]:

1. Gas bubble removal at the electrode surface
2. Electrolyte and electrode degassing
3. Disruption and thinning of the *Nernst* diffusion layer (*δ*)
4. Enhancement of mass transport of electroactive species through the double layer
5. Activation and cleaning of the electrode surface

**Fig. 13:**
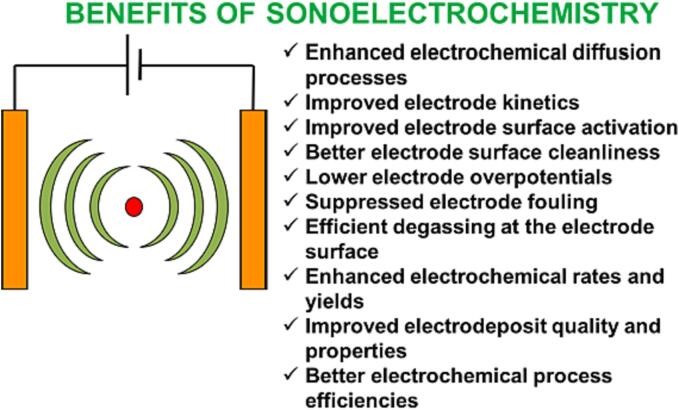
Benefits of sonoelectrochemistry.

**Fig. 14:**
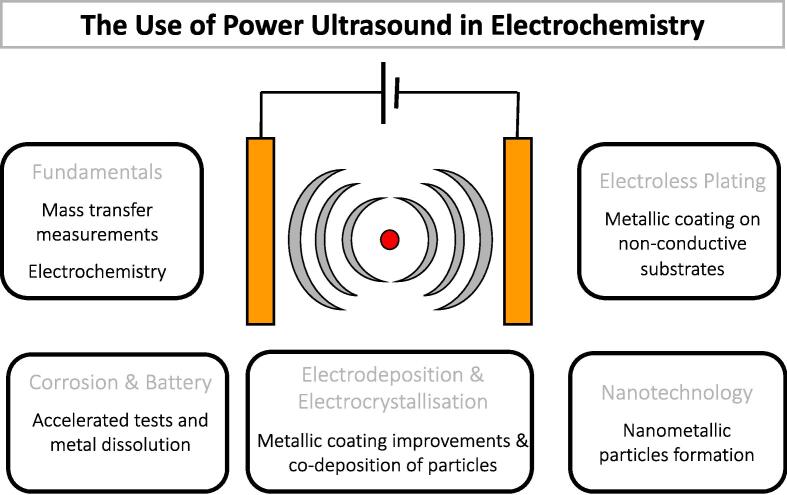
The use of power US in electrochemistry.

Fig. 13 shows the other benefits of sonoelectrochemistry, Fig. 14 exhibits the use of power US in electrochemistry, and Table 2 illustrates a list of major influencing factors of power US in electrochemistry [170].

**Table 2:** **Major influencing factors of power US on electrochemistry (modified from**[171]**).**

Influencing factors of power US on electrochemistry					
	**Acoustic streaming**	**Turbulent flow**	**Microjets and Microstreaming**	**Shock waves**	**Chemical** **effects**
Cause	• The power of acoustic streaming is directly proportional to the intensity of US, the surface area of the ultrasonic emitting device and the attenuation coefficient of the medium. It is inversely proportional to the bulk solution viscosity and the speed of sound.	• The movement of the acoustic cavitation bubbles.	• The collapsing of acoustic bubbles on a solid surface leads to the formation of microjets being directed towards the surface of the solid material at velocities of up to 200 m/s.	• Produced at the end of the strong collapse of the cavitation bubbles.	• “Sonolytic” effects in electrochemistry due to acoustic cavitation in the aqueous media.
Effect	• The enhancement of the movement of the solution. Reducing the diffusion boundary layer. Promoting the mass transfer of electroactive species to the electrode surface.	• Increases the mass transport process, like acoustic streaming, within the solution and at the electrode surface.	• If the surface is an electrode, the combined effects of the microjet and microstreaming enhance mass transport to the electrode surface. Electrode cleaning prevents fouling of the electrode surface (and accumulation of gas bubbles at the electrode surface) . Enhances the electrodeposition/electroplating processes.	• Erosion of the electrode surface leads to an increase in the local current.	• Formation of highly reactive radicals such as OH•, H•, H_2_O_2_•, and O• as well as molecular hydrogen peroxide.

**Fig. 15:**
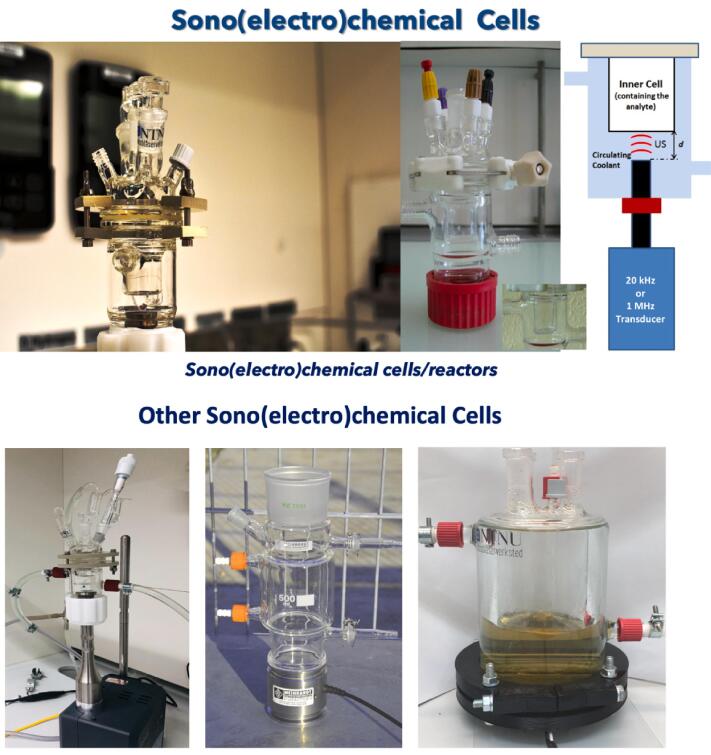
Various sono(electro)chemical reactors/cells.

Power US is usually transmitted using either an ultrasonic bath, an ultrasonic probe (horn) or an ultrasonic transducer (Fig. 15) [170], [171], [172], [173], [174], [101]. The ultrasonic probe and plate can either be directly immersed in the electrolyte or separated. An inner electrochemical cell is used when an ultrasonic probe or plate is separated from the electrolyte, as shown in Fig. 15. In both cases, the ultrasonic-emitting source should face the working electrode surface, known as the “face-on” geometry.

Despite its promising applications and benefits, sonoelectrochemistry has not been used widely in the industry and academia because scaling up can be an issue. However, this emerging area is recently gaining great attention because of the advances in ultrasonic equipment and modeling.

**Fig. 16:**
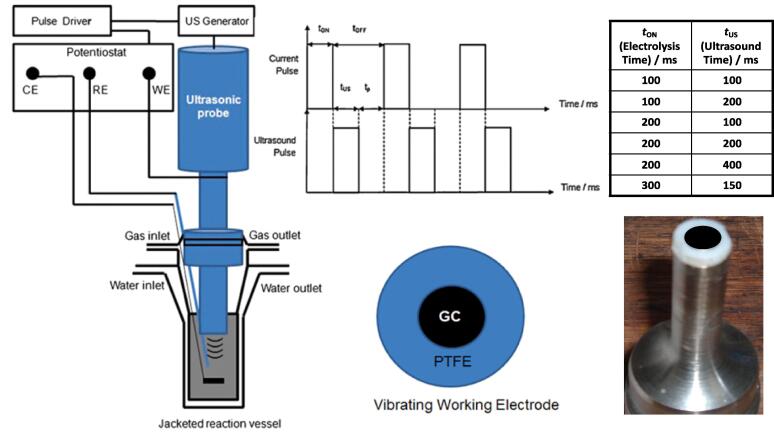
A schematic diagram of a sonoelectrochemical reactor set-up for producing nanomaterials [172], [101].

Recently, the use of US for producing energy materials, especially nanostructured materials for fuel cells, electrolysers, supercapacitors, and semiconductor catalysts, has provided many benefits regarding simplicity, efficiency, rapidity, and environmental friendliness [171]. Also, several investigations have demonstrated that the shape and size of the nanoparticles can be easily controlled by ultrasonication time, power, and frequency using either the sonoelectrochemical reactor set-up shown in Fig. 15 or 16 [170], [171], [172], [173], [174], [101].

For further information on using US in electrochemistry for energy and environmental applications, the readers are advised to check the recent reviews [171], [172], [173], [174], [101], [175], [176].

## 6 Scale-up challenges

The beneficial effects of cavitation, whether originating from ultrasonic or hydrodynamic sources, have resulted in many applications throughout the 20th century. US technology has been used for cutting, dispersing, welding, homogenizing, degassing, crushing, cleaning, drilling, degreasing processes, and flow measurements in various industries for decades.

Although sonochemistry has been studied and researched for several decades, it has not yet reached the same level of industrialization as other US technologies, such as cutting, welding, and cleaning. However, sonochemistry is still a very active and growing field of research with many discoveries and applications being explored, indicating a bright future for further R&D. Sonochemistry encompasses a wide range of applications, including polymerisation, crystallisation, cell disruption, atomisation, nanomaterial synthesis, drug delivery enhancement, processing, extraction, synthetic chemistry, and water treatment. These areas represent some of the most prominent and actively pursued research topics. The era of early discovery and exploration of sonochemistry in the 1980 s and 1990 s has now passed, and much knowledge has been accumulated over several decades. Nevertheless, the researchers of that period made significant contributions to the widespread use of US in the chemical sciences, benefiting both industry and academia.

Furthermore, the emergence of the Green Chemistry concept in the late 1990s, which promotes the use of environmentally-friendly technologies and processes, has increased the interest of the non-expert research and development community in sonochemistry. This is evident in the growing number of publications related to this field. The concept of Green Chemistry aims to reduce the global environmental impact of chemical transformations by minimizing energy consumption, shortening reaction times, reducing the use of hazardous and polluting substances, and promoting the reuse or valorization of waste materials [177]. There are 12 + 12 principles of Green Chemistry and Green Engineering (acronymic of IMPROVEMENTS and PRODUCTIVITY, respectively) focusing on designing reliable and sustainable chemical sciences for a cleaner world [177], [178].

**Fig. 17:**
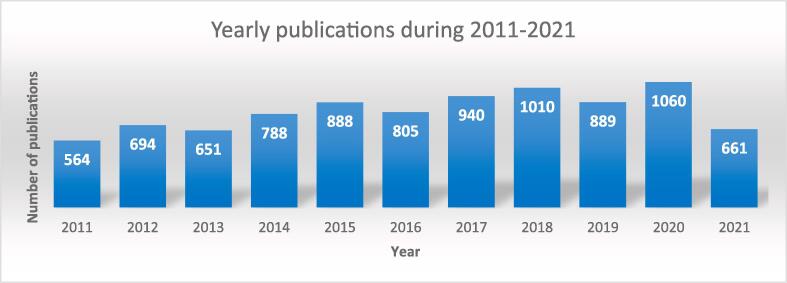
Google Scholar research on publications containing “sonochemistry” “sonochemical” or “ultrasound-assisted” words.

US is among the forefront technologies capable of accelerating, streamlining, and reducing the cost of chemical reactions without additional chemicals. This is due to the physical phenomenon of cavitation, which produces chemical and/or physical effects on the matter in the liquid phase. Furthermore, it enables chemical transformations to be carried out more cleanly and sustainably, in line with the principles of Green Chemistry. Low-frequency ultrasonic devices were first implemented and used because of the generated strong and harsh mechanical effects (accompanied by some weaker chemical effects) with accessible and cheap technology [179]. Commercially available ultrasonic devices typically come in the form of ultrasonic baths and probes, which can range in volume from a few milliliters to a few liters and operate in batch mode. Some continuous systems have recently been developed to treat larger volumes, indicating the potential for scaling up sonochemical processes to pilot and industrial scales. High-frequency US has been found to have great potential in various lab-scale applications such as wastewater treatment, synthetic chemistry, emulsion, crystallization, and enzymatic catalysis due to its physical and chemical effects. Therefore, it is important not to overlook the benefits of high-frequency US in these areas. While high-frequency US has shown potential for various lab-scale applications, its use on a larger scale is limited due to the lack of power generated by current devices. This limits its potential for short- or middle-term industrial applications. Only a limited number of high-frequency devices are available commercially, and they are intended solely for use at the laboratory scale. Fig. 17 shows yearly publications during 2011–2021 through a Google Scholar search containing “sonochemistry” “sonochemical” or “ultrasound-assisted” words.

Scaling up US-assisted processes from lab scale to pilot or industrial scale is a desirable goal, but examples of successful scaling-up are rare in the literature. This suggests that the sonochemistry field still lacks the necessary technical and scientific maturity to tackle scaling-up challenges easily. What are the reasons for sonochemistry's struggle to overcome this significant technical obstacle? Many complex and multifaceted reasons explain why sonochemistry struggles to overcome this significant technical hurdle. While it is difficult to delve into these issues in detail, some key factors shed light on the challenges faced in this field. In order to scale up a sonochemical process, it is necessary to optimize the lab-scale process and carefully study, control and understand several key operating parameters. These parameters include incident frequency, acoustic energy, temperature, time, gas, and properties and ratios of products, solvents, and solutes [180], [181]. Optimizing the lab-scale process is a necessary but time-consuming step that requires studying, controlling, and understanding several key operating parameters. However, this step can be shortened by using mathematical and computational tools such as RSM or ANOVA, which can limit the number of experiments needed to measure the impact of the studied operating and experimental parameters on the intended process [182]. During scale-up, it is important to ensure that the quality and characteristics of the final product remain consistent with the lab-scale optimization phase while increasing productivity.

One of the critical factors for successful scale-up in sonochemistry is achieving adequate reactor design coupled with energetically-efficient US emitters. This is essential to ensure a homogeneous cavitational field and optimum acoustic streaming throughout the reactor, which can help achieve the desired product characteristics and increase productivity. Studies attempting to scale up an existing lab-scale sonochemical process have revealed that the non-uniformity of the ultrasonic field and the achievement of optimal acoustic streaming are critical factors that need to be addressed to increase the scale of the reaction [183], [184], [185]. Over the past 30 years, the sonochemistry community has developed various characterization methodologies to characterize cavitational activity in the reactor fully. These include sonoluminescence, calorimetric measurement, impedance measurement, acoustic pressure mapping, dosimetries (such as Fricke, Weissler, terephthalic acid, salicylic acid, iodometry, etc.), temperature mapping, and hydrophone measurement. These methods help understand the nature of the cavitational activity and ensure that it is homogeneous and optimal throughout the reactor [186], [187], [188], [189], [190]. The most efficient characterization tools are suitable for batch US systems at any suitable working frequency on probes or cup-horn technologies for laboratory scale operation. The characterisation methodologies indicated above have demonstrated that cavitational activity in US systems can be heterogeneous, with limited power dissipation, which makes it difficult to increase the scale of the process. This suggests that batch ultrasonic systems may not be the best approach for designing pilot or industrial scale ultrasonic processes. Another challenge related to the use of low-frequency probes is the overheating of transducers. This issue is connected to the limited capability of most commercially available low-frequency probes to sustain high acoustic amplitude for an extended period, regardless of their size. Additionally, tip erosion of the probe can occur, leading to contamination and further complications.

Ever since its establishment by Professor T.J. Mason in 1994, the Ultrasonics Sonochemistry journal has been at the forefront of the field of sonochemistry, making it a dynamic and engaging domain with regular publications, as well as occasional Special Issues that delve into the obstacles, difficulties, and prospects of US applications in research and development. The articles published in the special issue titled “Sonochemistry: Scale-up and industrial development,” managed by Prof. C. Pétrier in 2010, provided valuable perspectives on the scientific, technical, and economic aspects of scaling up sonochemical processes in various applications, such as wastewater treatment, cleaning, bio-waste extraction and valorisation, oxidative desulfurisation, reactor characterisation, novel ultrasonic emitting technology, leather processing, bacterial disinfection, and dairy processing [191]. Many researchers have reported that scaling up an ultrasonic process is highly feasible. However, several technical hurdles have to be crossed over, notably in terms of energetic consumption, design of ultrasonic reactors and location/nature/design of emitters to ensure the homogeneity of the dispersed chemical and/or physical effects of US throughout the reactor. Flow mode also appeared as a valuable and cost-effective alternative to batch mode to increase treated volumes without scaling up the ultrasonic device, leading to controlled energetic consumption.

The main goal in sonochemistry is to scale up ultrasonic systems. The scientific community has recognized that working in a flow mode can facilitate the scale-up of such processes without significantly increasing the required ultrasonic power. In 2014, Peshkovsky et al. demonstrated that by using a continuous-flow approach with their original ultrasonic Barbel horn, which allowed for high-amplitude ultrasonic power with a treated flow of 4 L/min, the energetic demand of the ultrasonic processor was only 3 kW. This was compared to the microfluidiser technology, which required 37 kW to achieve a similar result of D_50_ [192]. While flow chemistry is a valuable solution for reducing the energetic demand of ultrasonic processes during scaling up, it is not always a viable option. Sometimes, using the flow mode is impossible, and the batch mode remains the only viable alternative. Allen et al. investigated using ultrasonic technology to accelerate the slow process of pork curing, which is hindered by poor mass transfer. They used a 55 L batch reactor with ceramics at the bottom and two ultrasonic probes at the top since the ultrasonic batch did not feature a variation of the ultrasonic power. With this combined system, they could reduce by 50% the usual time required to salt meat samples, regardless of the ultrasonic intensity used, while maintaining the quality and characteristics of the meat samples [193]. The authors suggested that this approach could be applied in industrial meat-curing processes but acknowledged that energy and cost analyses would need to be conducted before implementation. Contrary to several other fields where ultrasonic energy demand is high, such as environmental sciences, synthesis chemistry, or cleaning, food processing requires low-energy processes to avoid altering the quality and attributes of the food. As a result, the batch mode can still be used in some food processing steps. In 2017, Paniwnyk published a comprehensive review on the use of US technology for processing liquid food such as beverages, dairy, drinks, etc. The review included several pilot-scale examples. Both batch and continuous flow systems have been developed, often with customized ultrasonic transducers and reactors capable of treating volumes ranging from a few liters to 1 cubic meter, highlighting the increasing interest of companies in utilizing US for food processing applications [154]. The successful pilot-scale implementations discussed in the review are presented in Table 3. Table 4 presents the selected recent articles/reviews focusing on the implementation of continuous-flow US technologies.

**Table 3:** Selected examples of ultrasonic pilot-scale food processing.

**Reference**	**Purpose**	**Operating conditions**
[194]	Malaxation of olive oil	Ultrasonic bath, 35 kHz, 150 W, 4.25 L
[195]	Nanoemulsions of coconut oil	Batch mode, 20 kHz (750 W, 1 kW and 2 kW), 2L
[196]	Reconstituted whey protein in dairy	Flow mode, 20 kHz, 4 kW, 0.2 to 6 L
[197]	Sterilisation and microbial control of fruit/vegetable juices	Batch mode, combination with UV, 20 kHz, 100 W, 5 L
[198]	Extraction of saccharides from 28% w/v corn slurry	20 kHz, 3.3 kW Batch mode: 20–40 s Continuous mode: 10–28 L/min

**Table 4:** Selected recent articles/reviews exploring the implementation of continuous-flow US technologies.

Ref	Title
[205]	Ultrasound assisted continuous processing in microreactors with focus on crystallization and chemical synthesis: A critical review
[206]	Recent progress on ultrasound-assisted electrochemical processes: A review on mechanism, reactor strategies, and applications for wastewater treatment
[207]	Laboratory evaluation to field application of ultrasound: A state-of-the-art review on the effect of ultrasonication on enhanced oil recovery mechanisms
[208]	Review of ultrasound combinations with hybrid and innovative techniques for extraction and processing of food and natural products
[209]	Recent Advances in the Application of Enzyme Processing Assisted by Ultrasound in Agri-Foods: A Review
[210]	Continuous Ultrasonic Reactors, Design, Mechanism and Application
[211]	Recent development in high quality drying of fruits and vegetables assisted by ultrasound: A review
[212]	Industrial Ultrasound Applications in the extra-virgin olive oil extraction processes: History, Approaches, and Key questions
[213]	Recent advances in the application of ultrasound in dairy products: Effect on functional, physical, chemical, microbiological and sensory properties

In the same year, Gogate et al. published a review focusing on the engineering aspects of US to improve chemical syntheses [199]. US has been shown to enhance the reactivity of numerous chemical reactions, whether conducted under homogeneous or heterogeneous conditions, utilizing its physical or chemical effects, or a combination of both. The review not only discusses the latest advancements in US to enhance chemical reactions but also covers engineering aspects related to scaling up the process, along with providing some literature examples. The review's authors emphasized the importance of using multiple transducers in a well-designed ultrasonic reactor to achieve an intense and uniform dissipation of ultrasonic power and cavitational activity throughout the reactor for large-scale processes ranging from a few mL to a few hundred L. The authors provided several examples of attempts to scale up the use of US, with most of them being in batch mode and involving using multiple transducers to meet the increased scale of work. This may be due to the high cost of chemicals, catalysts, and solvents or because using ultrasonic flow designs was not yet considered a viable alternative to control overall energy demand while preserving ultrasonic efficiency. Furthermore, it appears that the energetic considerations were not considered, as multiplying the number of transducers can be counterproductive for scaling up due to the increase in the number of irradiating transducers. The authors concluded that while there have been some interesting attempts to scale up organic synthetic chemistry processes using US, the number of such attempts is still relatively small. The authors suggested that a multidisciplinary approach involving theoretical modelling, material science, and chemical engineering should be taken to scale up lab-scale processes using ultrasonic technology effectively. In addition, they emphasized the importance of optimizing typical ultrasonic parameters such as frequency, power, and time, as well as reactor design, to achieve successful scaling up. To this end, they proposed some guidelines to tune these important operating parameters to maximize the ultrasonic effects. The use of low-frequency US in sonochemistry has made the extraction of bioactive molecules a highly researched area due to the intense physical effects it produces.

The literature contains a plethora of research on the use of low-cost and straightforward ultrasonic techniques, both in batch and continuous modes, for the extraction of valuable bioactive compounds from various sources of biomass feedstocks such as barks, leaves, stems, shells, cocoons, powders, slurries, and waste materials. This reflects the perception of US as an affordable, user-friendly, and readily available technology for developing sustainable chemistry. Manickam’s group researched the US-assisted extraction (UAE) of β-D-glucan polysaccharides from Ganoderma ludidum, a fungus known to contain more than 400 bioactive molecules with various pharmaceutical activities. The study aimed to increase the extraction yield of the polysaccharides while reducing the extraction time and solvent consumption [200]. Following the optimization of their process in the lab-scale and batch mode (using a 250 mL volume and 10 mg of feedstock) through RSM and statistical analysis, the authors proceeded to investigate the scalability of their method by increasing the treated volume from 250 mL to 1, 2, 3, 4, and 6 L. The authors noted that their ultrasonic system could not maintain extraction efficiency as they increased the treated volume in batch mode. However, they found a solution to this problem by modifying their process to a flow mode when scaling up to 3 and 6 L. Chemat et al. conducted a comparative study on the UAE of clove buds in both batch and continuous modes, and found that the continuous mode was more suitable for scaling up due to its ability to control the energetic demand [201]. Cravotto et al. also extracted polyphenols from grape stalks using both batch and continuous modes [202].

Waste from winemaking is a global issue that has a considerable impact and leads to high disposal costs. However, these wastes also contain valuable biomolecules such as sterols, triterpenes, polyphenols, stilbenes, phenolic acids, and more, which positively affect human health. Indeed, this example highlights the potential of extracting valuable bioactive molecules from winemaking wastes to reduce disposal costs and environmental impact. By doing so, the waste can be valorised, and the extracted biomolecules can be used in various industries such as food, pharmaceuticals, and cosmetics, among others, which can also benefit from using sustainable and eco-friendly processes. The authors conducted a preliminary UAE experiment at the laboratory scale using a classic immersed horn and a cup-horn system operating at 21 and 25 kHz, respectively, to determine the optimal operating parameters before scaling up the process. The authors used a loop design for the continuous mode, which involved a 15 L square reactor fitted with multiple low-frequency piezo-ceramics emitting at 29 kHz (2 kW). The reactor was connected to a 120 L tank equipped with a 4-bladed impeller to ensure proper mixing and suspension of the solid waste in the water. The authors made various technical and engineering modifications to scale up their process while minimizing any losses in yield and activity of the extracted molecules. This recent example shows that scaling up a lab-scale process with US is no longer a far-fetched idea but a current reality. Moreover, Luo et al. have investigated the UAE of anthocyanin cyanidin-3-galactoside (Cy3-gal) from the peels of *Pyrus Communis* fruit [203]. Similar to the earlier study by Cravotto et al., the authors conducted laboratory-scale experiments to determine the optimized operating parameters before scaling up the process by designing a loop/continuous system. The authors concluded their study by stating that, despite yielding less anthocyanin than the batch mode (0.285 mg/g versus 0.315 mg/g, respectively), the flow system's efficiency could be improved through further optimization of instrumentation design and process factors. Nanoemulsions are another example where the scaling-up of US technology can be extremely advantageous. Nanoemulsions are utilized in various applications spanning different industries, such as chemicals, paints and coatings, cosmetics, pharmaceuticals, and food. Several technologies, such as high-pressure homogenizers, rotor–stator systems, and conventional stirrers, are available for producing these emulsions. However, most of these methods tend to be energy-intensive and time-consuming. Compared to these methods, US has several advantages, such as producing a narrow particle size distribution, providing time-stable emulsions, and requiring low energy input.

**Fig. 18:**
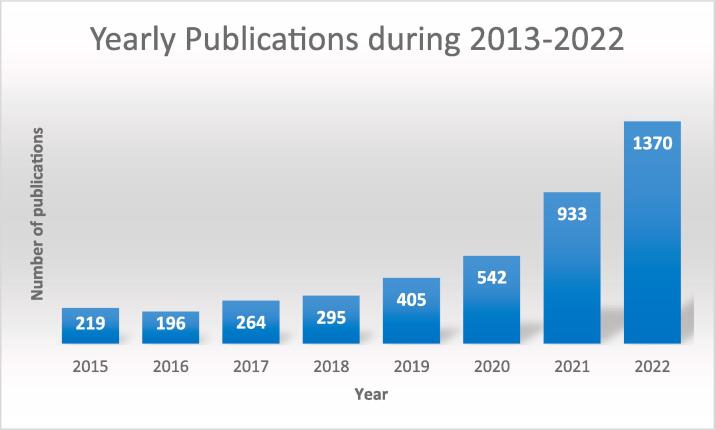
Google Scholar research on publications containing “sonochemistry”, “ultrasound” and “continuous” words.

Efforts to scale ultrasonic-assisted processes have been the subject of intense investigation to overcome technical challenges and transition lab-scale processes to industrial pilot processes. In the R&D community, two consensus approaches are gradually emerging for achieving an acceptable working scale for industrial applications. The first approach focuses solely on US technology. It employs various means such as US/US coupling, increased number of US sources, addition of chemicals to enhance reactivity, modification of pressure/temperature/gas atmosphere, and transitioning from batch mode to flow mode. This field has been actively growing for decades and shows promising advancements. The second approach explores integrating other technologies, such as micro-flow technology, microwave (MW) heating, photochemistry, atomization, hydrodynamic cavitation, etc. These technologies are sought to provide complementary, additional, or synergistic effects to those of US. While this field is relatively newer, recent articles and the release of a Special Issue in 2022 by Ultrasonics Sonochemistry journal dedicated to US-hybridized technologies in several chemical sciences domains highlight this combined technology's growing interest and potential. It is important to note that the road to developing an effective and scalable process can be lengthy and requires further research and development. Many currently explored systems are still prototypes, often built unconventionally with available equipment. Implementing a pilot or industrial-scale process may take time [204]. Nevertheless, the field focusing solely on US technology, particularly in continuous flow sonochemistry, remains the most explored and active area of research in terms of scaling up ultrasonic-assisted processes. Fig. 18 in this review illustrates the active research landscape of continuous flow sonochemistry, showcasing its significant presence and ongoing developments based on a survey conducted on Google Scholar.

This significant progression observed, especially from 2019 onwards, should be considered with the simultaneous increase in publications on US-hybridized technologies. This explosion of research aligns with the accelerating trend depicted in Fig. 18 since 2018. While the number of publications on flow or continuous US is impressive, it is important to note that the field of US-hybridized technologies has also seen substantial growth since 2019. This indicates a rising interest and exploration of new technological approaches in sonochemistry and related topics, particularly concerning industrial applications. Listing all the recent articles and reviews in this area would be extensive, as there have been numerous contributions in recent years. However, some selected examples of recent articles or reviews provide insights into the current developments in continuous US technology.

It is worth mentioning that the focus of publications in continuous US is often not solely on this specific technology but rather on comparing batch and continuous US approaches. These comparisons may also involve adding another technology to achieve additive or synergistic effects. As indicated earlier, two main scaling routes have been identified: the “classical sonochemical way” and the combination of US with another technology. Recent articles in the field demonstrate that these two paths are gradually converging. The R&D community has come to understand that scaling up US technology will likely involve one or both of these approaches consecutively or simultaneously.

## CRediT authorship contribution statement

**Sivakumar Manickam:** Writing – original draft, Writing – review & editing. **Daria Camilla Boffito:** Writing – original draft, Writing – review & editing. **Erico M.M. Flores:** Writing – original draft, Writing – review & editing. **Jean-Marc Leveque:** Writing – original draft, Writing – review & editing. **Rachel Pflieger:** Writing – original draft, Writing – review & editing. **Bruno G. Pollet:** Writing – original draft, Writing – review & editing. **Muthupandian Ashokkumar:** Writing – original draft, Writing – review & editing.

## Declaration of Competing Interest

The authors declare that they have no known competing financial interests or personal relationships that could have appeared to influence the work reported in this paper.
